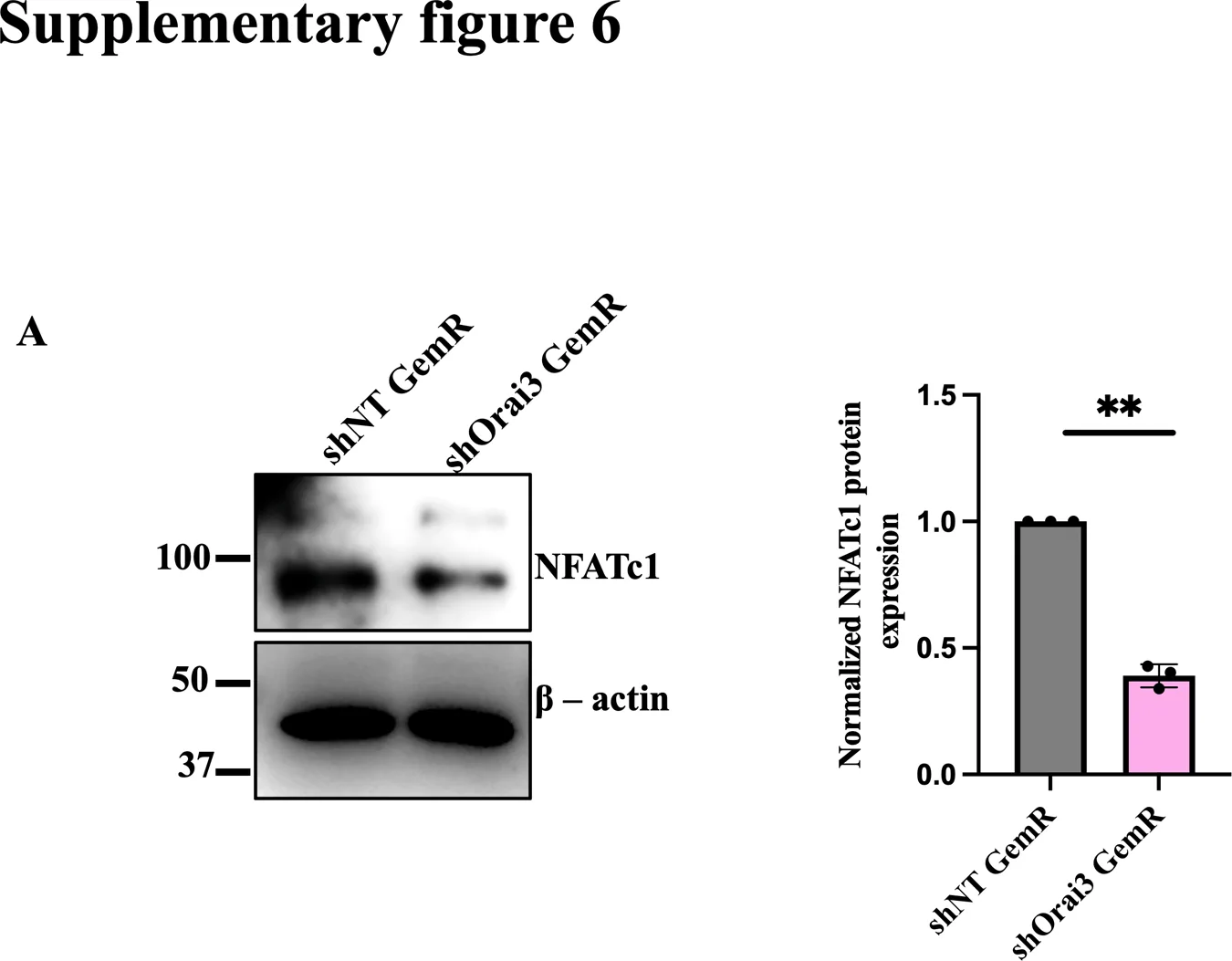# The Ca^2+^ channel Orai3 drives gemcitabine resistance in pancreatic cancer through NFATc1-dependent SLIT3 expression

**DOI:** 10.1016/j.jbc.2026.113480

**Published:** 2026-08-25

**Authors:** Samriddhi Arora, Abhishek Tanwar, Gyan Ranjan, Rajender K. Motiani

**Affiliations:** Laboratory of Calciomics and Systemic Pathophysiology (LCSP), Regional Centre for Biotechnology (RCB), Faridabad, Delhi-NCR, India

**Keywords:** Orai3, store operated calcium entry, pancreatic cancer, chemoresistance, Ca^2+^ signaling, NFATc1, SLIT3

## Abstract

Pancreatic Cancer (PC) is one of the most aggressive cancers and is associated with poor prognosis. One of the routinely used chemotherapeutic drugs for the treatment of advanced PC is gemcitabine, which offers survival benefits to patients. However, acquired gemcitabine resistance leads to recurrence and metastasis, and the long-term prognosis remains poor. Although there is substantial clinical evidence of gemcitabine resistance, the cellular and molecular mechanisms that drive this resistance remain largely unappreciated. Here, we reveal that Orai3, a Ca^2+-^selective channel, is a crucial inducer of gemcitabine resistance. We demonstrate that Orai3 is overexpressed and hyperfunctional in gemcitabine-resistant PC cells. Orai3 silencing in gemcitabine-resistant cells enhances chemo-sensitivity, inhibits migration, stalls invasion, and moderates stemness characteristics. Notably, studies in the zebrafish model corroborate Orai3’s significance in gemcitabine resistance *in vivo*. Mechanistically, our unbiased RNA-seq analysis, coupled with robust functional studies, reveals that a secretory repulsive axon guidance molecule, Slit guidance ligand 3 (SLIT3), acts downstream of Orai3 to induce gemcitabine resistance. Finally, we report that the Nuclear Factor of Activated T cells 1 (NFATc1) transcription factor bridges Orai3 to SLIT3 transcription. Taken together, this study reveals Orai3 as a promising target to combat gemcitabine resistance and uncovers a unique Orai3-NFATc1-SLIT3 signaling module that drives chemoresistance.

Pancreatic Cancer (PC) is one of the leading causes of cancer-associated deaths worldwide, with a 5-year survival rate of around 10% (1, 2). PC is likely to surpass breast, prostate, and colorectal cancers to become the second leading cause of cancer-related death by 2040 (3). The primary reasons for high mortality in PC are metastasis and chemoresistance. Currently, gemcitabine is one of the chemotherapeutic drugs that is given either alone or in combination for treating advanced and metastatic PC (4, 5). It is a deoxycytidine analogue inhibiting DNA replication and preventing tumor growth (4). Despite being the cornerstone of PC treatment for the last 2 decades, patients develop gemcitabine resistance within weeks of treatment initiation (6). Hence, there is an urgent need to overcome gemcitabine resistance by identifying the underlying mechanisms and targeting them to enhance gemcitabine efficacy (7).

Emerging literature suggests that dysregulated Ca^2+^ signaling plays an important role in chemoresistance (8, 9). Further, STIM1, an ER Ca^2+^ sensor, is reported to contribute to gemcitabine resistance in PC (10, 11). STIM1 senses endoplasmic reticulum (ER) Ca^2+,^ and upon depletion of Ca^2+^ in the ER, it activates plasma membrane channels to bring Ca^2+^ into the cytosol. Since ER Ca^2+^ stores regulate intracellular Ca^2+^ influx, this process is known as Store-operated calcium entry (SOCE). SOCE leads to increases in the cytosolic Ca^2+^ concentration required for several cellular processes, including cell proliferation, migration, differentiation, and apoptosis. Furthermore, SOCE contributes to enhanced migration and invasion in cancer cells. Importantly, elevated expression of SOCE players is associated with poor prognosis in a variety of cancer types (12, 13, 14, 15). Although there is some evidence supporting the role of STIM1 in chemoresistance, the identity of Ca^2+^ influx channel responsible for driving gemcitabine resistance and the underlying molecular mechanism remains unappreciated. Moreover, there is limited information on targetable molecular players that contribute to acquired gemcitabine resistance, which presents a significant challenge for patients undergoing chemotherapy.

We here reveal that the Orai3 Ca^2+^ channel plays a critical role in gemcitabine resistance *via* SLIT3 signaling. We generated stable gemcitabine-resistant cell lines derived from their sensitive parental line by subjecting them to progressive gemcitabine exposure. We analyzed the expression of key Ca^2+^ handling proteins in them and found elevated Orai3 and STIM1 levels. To determine the role of Orai3 in chemoresistance, we conducted live-cell Ca^2+^ imaging, a variety of cellular assays and *in vivo* studies. These studies demonstrated that Orai3 drives gemcitabine resistance and contributes to cancer cell stemness. Moreover, we uncovered Orai3’s role in regulating the metastatic potential of gemcitabine-resistant cells *in vivo* by performing live animal imaging in a zebrafish model. Mechanistically, our unbiased transcriptomics revealed an interesting differential regulation of SLIT3 downstream of Orai3 in gemcitabine-resistant cells. We performed SLIT3 gain-of-function as well as loss-of-function studies and identified a crucial oncogenic role of SLIT3 in the progression of gemcitabine-resistant PC. Our data demonstrate that downstream of Orai3, SLIT3 regulates cell proliferation, migration, invasion, and stemness of gemcitabine-resistant PC cells. Finally, we identified that the NFATc1 transcription factor connects Orai3 to SLIT3 transcription. Taken together, this study advances our understanding of the molecular mechanisms driving chemoresistance in PC. Importantly, it identifies Orai3 as a promising therapeutic target to overcome gemcitabine resistance.

## Results

### Generation and characterization of gemcitabine-resistant cell lines

To delineate the role of Ca^2+^ signaling in the development of PC chemoresistance, we first established stable gemcitabine-resistant (GemR) pancreatic cancer cell lines, *i.e.*, CFPAC-1 and AsPC-1 GemR, from the parental CFPAC-1 and AsPC-1 cell lines by exposing them to increasing doses of gemcitabine, as shown in the schematic diagram (Fig. 1*A*). Cells were considered GemR once the maximal inhibitory concentration was significantly higher in GemR cells than in parental cells. The IC_50_ values of GemR cells showed an increase compared to parental cell lines (CFPAC-1: 1.91 μM *versus* CFPAC-1 GemR: 36.65 μM and AsPC-1: 2.035 μM *versus* AsPC-1 GemR: 12.28 μM) (Fig. 1*B*). The transporters and enzymes that metabolize gemcitabine drive acquired GemR in human pancreatic cancer are regarded as a predictive marker for response to gemcitabine in clinical settings. To validate the acquired resistance in our GemR cell lines, we quantified the expression of such well-established markers of GemR (6, 16), *that is,* human equilibrative nucleoside transporter-1 (*hENT1*), deoxycytidine Kinase (*dCK*), Ribonucleotide reductase 1 (*RRM1*), and Ribonucleotide reductase 2 (*RRM2*). Our investigation showed significant differential expression of these markers in the two gemcitabine-resistant cell lines (Fig. 1*C*). Further, as reported earlier, the acquired resistance in the cell lines was estimated using the ratio of *hENT1∗ dCK/RRM1∗ RRM2* (17). Calculation of the relative expression ratio using the established formula showed a consistently lower ratio in both cell lines, suggesting establishment of chemoresistance (Fig. 1*D*). Our findings demonstrated that both CFPAC-1 GemR and AsPC-1 GemR have acquired gemcitabine resistance, as evidenced by the increased IC_50_ values and expression of gemcitabine resistance-specific markers.

**Figure 1:**
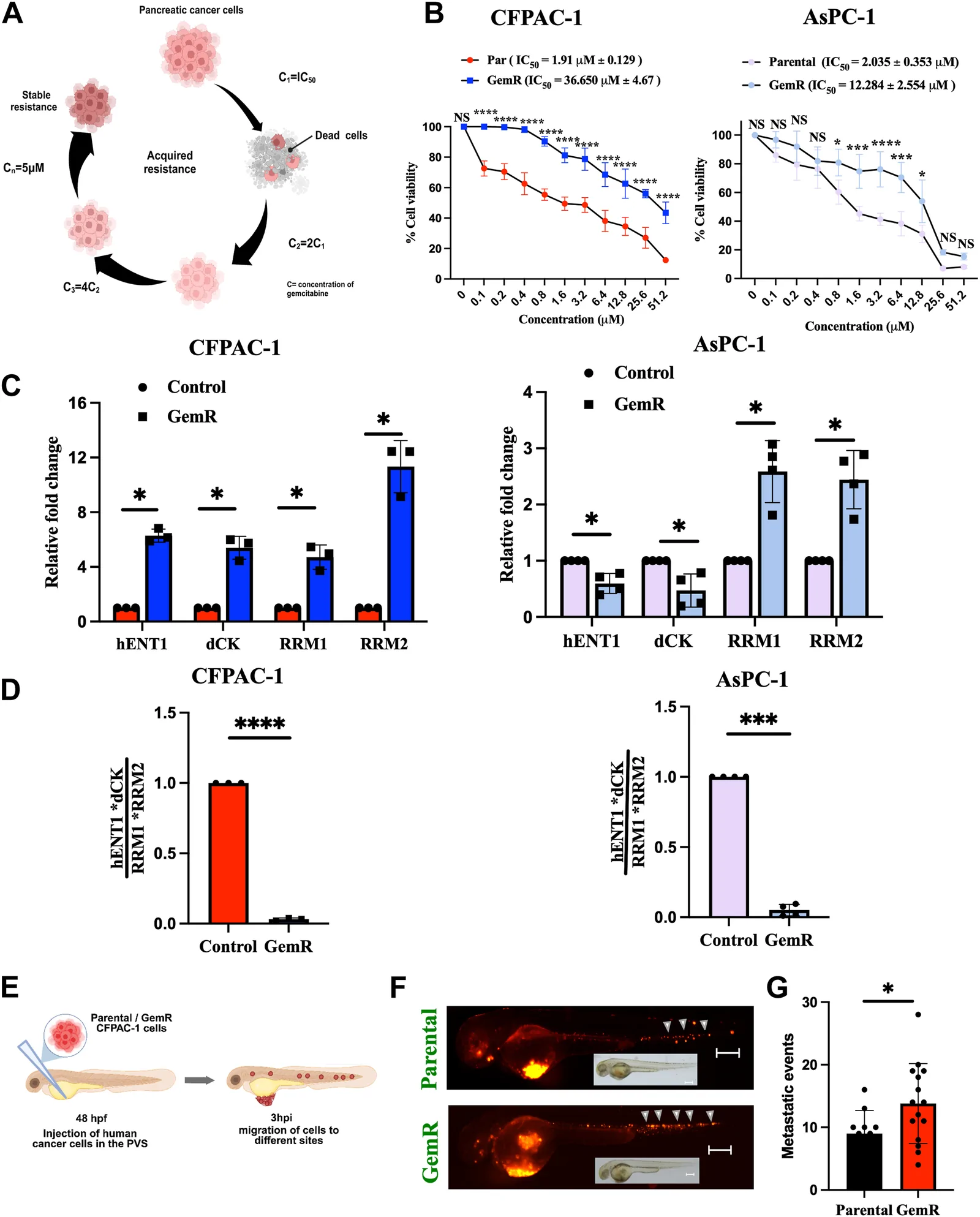
**Generation and characterization of gemcitabine-resistant cells.***A*, diagram illustrating the development of acquired gemcitabine-resistant cells. *B*, MTT assay showing IC50 values of Parental *versus* GemR in CFPAC-1 and AsPC-1 cell lines. treated with gemcitabine for 72 h. The absorbance was normalized to the respective no-treatment absorbance (N = 3). ∗∗∗∗*p* < 0.0001 (CFPAC-1), ∗*p* = 0.0277, ∗∗∗*p* = 0.0007, ∗∗∗∗*p* < 0.0001, ∗∗∗*p* = 0.0002 and ∗*p* = 0.0126 (AsPC-1). *C*, mRNA expression of *hENT1, dCK, RRM1,* and *RRM2* relative to *GAPDH* of GemR *versus* parental in CFPAC-1 and AsPC-1 cell lines (N = 3). ∗*p =* 0.0027, ∗*p =* 0.0117, ∗*p =* 0.0187, ∗*p =* 0.0111 (CFPAC-1), ∗*p =* 0.0198, ∗*p =* 0.0367, ∗*p =* 0.0104, ∗*p =* 0.0115 (AsPC-1). *D*, Validation of gemcitabine resistance by *hENT1 x dCK/RRM1 x RRM2* expression ratio in GemR CFPAC-1 and AsPC-1 cells. The value of the ratio in parental cells was set as 1.0 (N = 3). ∗∗∗∗*p* < 0.0001 (CFPAC-1), ∗∗∗∗*p* < 0.0001 (AsPC-1). *E*, scheme representing the approach to inject pancreatic cancer cells (Parental, GemR, shNT, and shOrai3 GemR) in the Perivitelline Space (PVS) of 48 hpf *Assam wild type (Aswt)* zebrafish larvae. *F*, representative images of zebrafish larvae 3 h post-injection with parental and GemR labeled cells (*red*) captured using a stereomicroscope. Scale bar: 500 μm. *G*, quantitative analysis of the metastatic capacity of CFPAC-1 parental and GemR cells upon engraftment into zebrafish (n = 15). ∗*p* < 0.0359 (CFPAC-1) Data presented are mean ± SD. For statistical analysis, 2-way ANOVA was performed, followed by Šídák's multiple comparisons test for *panel B*. One-sample *t* test was performed for *panels C* and *D*, and an unpaired *t* test was performed for *panel G* using GraphPad Prism software. Here, ∗*p* < 0.05; ∗∗*p* < 0.01; ∗∗∗*p* < 0.001 and ∗∗∗∗*p* < 0.0001.

GemR pancreatic cancer is more aggressive, and it is associated with higher metastatic potential (16, 18). Therefore, to assess the metastatic aggressiveness associated with GemR, we performed *in vivo* xenograft studies in zebrafish (*Danio rerio*). As shown in the schematic diagram (Fig. 1*E*), we injected parental and GemR CFPAC-1 cells in the perivitelline space of 48 h post-fertilization (hpf) old larvae. As reported earlier, the migration of cells at different sites post-injection was used to evaluate the metastatic potential of these cells (19, 20). We observed that CFPAC-1 GemR cells exhibited more aggressive metastatic behavior than the parental cells (Fig. 1, *F* and *G*). This observation confirmed the successful generation of a GemR cell line model with an enhanced metastatic ability as reported in pancreatic cancer patients.

### Orai3 expression and function is augmented in GemR cells

Earlier studies have shown a strong correlation between the expression of STIM proteins and chemoresistance (21). We earlier reported that Orai3 encodes a functional SOCE channel in PC cells (15). However, the role of Orai proteins in acquired PC chemoresistance remains unknown. Therefore, we first unbiasedly examined the expression of Orai and STIM proteins in parental and GemR cells. Interestingly, only Orai3 and STIM1 showed significant upregulation of expression in both CFPAC-1 GemR (Fig. 2*A*) and AsPC-1 GemR (Fig. 2*B*) cells. In contrast, other paralogs showed either decreased or unchanged expression in GemR cells (Fig. 2, *A* and *B*). These data are also shown in the heatmap (Fig. S1, *A* and *B*). Each square in the heatmap represents the normalized densitometry values for the respective protein bands, normalized to their corresponding beta-actin loading controls. These values reflect the expression levels of Orai1, Orai2, Orai3, STIM1, and STIM2 proteins in control and corresponding GemR CFPAC-1 and AsPC-1 cells. Next, to investigate Orai3 activity in GemR cells, we performed live-cell Ca^2+^ imaging using Fura-2 AM. As reported in our previous study (15), SOCE was assessed using a standard thapsigargin (Tg) based protocol and 2APB treatment was given to distinguish between Orai1 and Orai3 (22, 23). The literature suggests that 2APB at 50 μM activates Orai3 and inhibits Orai1 (23, 24, 25). Upon Tg-mediated ER Ca^2+^ store depletion, SOCE was studied by adding external Ca^2+^. Notably, as shown in representative Ca^2+^ imaging traces, we observed a significant increase in SOCE and 2APB potentiation in CFPAC-1 GemR (Fig. 2*C*) and AsPC-1 GemR cells (Fig. 2*D*) compared to their parental cells. Further, Ca^2+^ entry and 2APB potentiation were calculated for individual cells in each dish and they were significantly higher in both the GemR cell lines in comparison to their respective parental cells (Fig. 2, *E* and *F*). This Ca^2+^ imaging data corroborates the protein expression data, suggesting that GemR augments SOCE by enhancing Orai3 and STIM1 expression.

**Figure 2:**
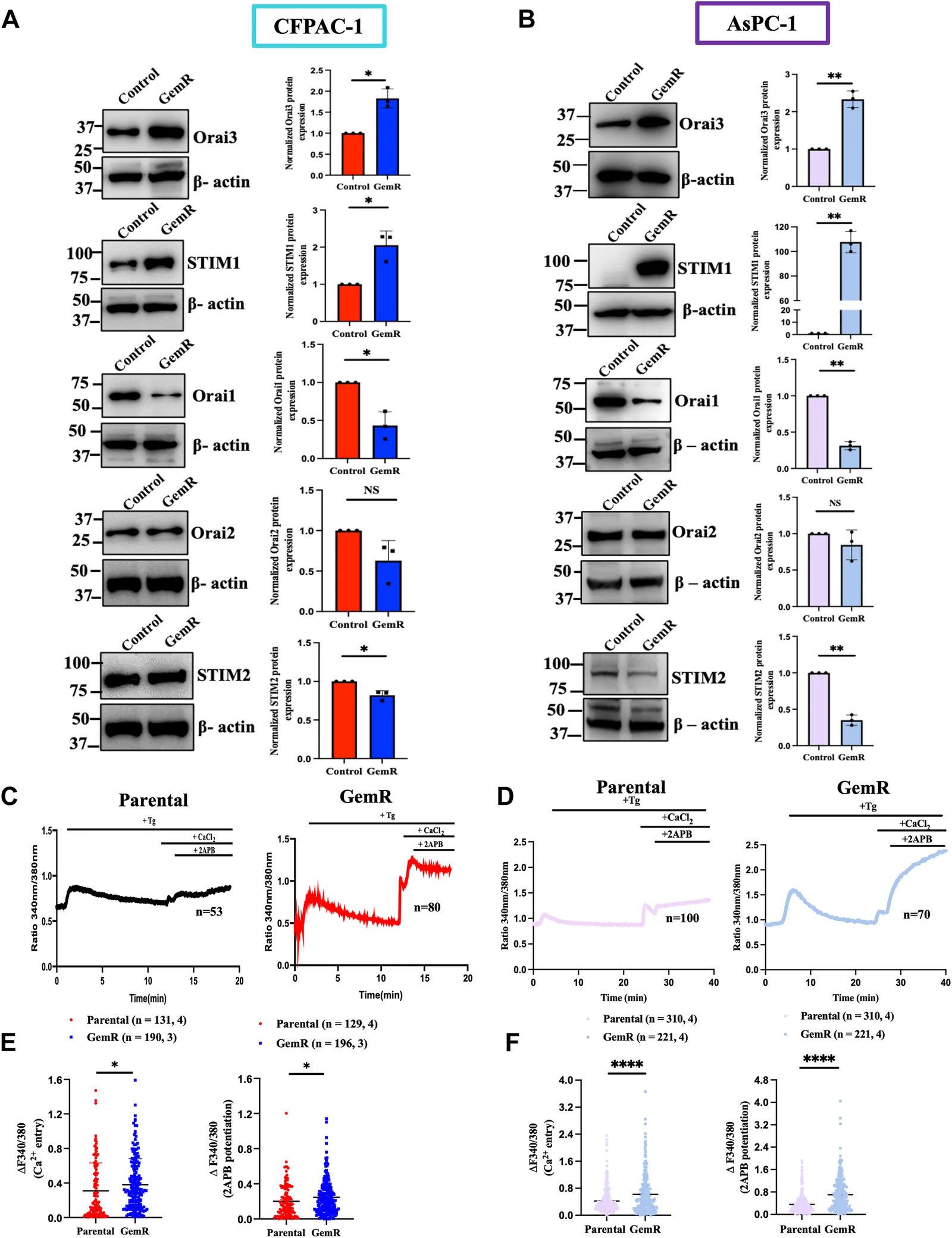
**Orai3 expression and function is augmented in GemR cells.***A*, representative Western blot and densitometry showing Orai3, STIM1, Orai1, Orai2, and STIM2 expression in Parental and GemR CFPAC-1 cells (N = 3). (Note: Orai2 and STIM2 were probed on the same membrane, so the same actin loading control is shown for both.) ∗*p* = 0.0247, ∗*p =* 0.0412, ∗*p* = 0.0327, ∗*p* = 0.0358 (CFPAC-1). *B*, representative Western blot and densitometry showing Orai3, STIM1, Orai1, Orai2, and STIM2 expression in Parental and GemR AsPC-1 cells. (N = 3). ∗∗*p* = 0.0094, ∗∗*p* = 0.0021, ∗∗*p* = 0.0021, ∗∗*p* = 0.0041 (AsPC-1). *C*, representative Ca^2+^ imaging traces of Parental and GemR CFPAC-1 cells, where “n” denotes the number of cells in the trace. *D*, representative Ca^2+^ imaging traces of Parental and GemR AsPC-1 cells, where “n” denotes the number of cells in the trace. *E*, quantitation of Ca^2+^ entry and 2APB potentiation in Parental and GemR CFPAC-1 cells where “n = x,y” where “x” denotes the number of ROI in each condition and “y” denotes the number of dishes recorded. ∗*p* = 0.0459, ∗*p* = 0.0451. *F*, quantitation of Ca^2+^ entry and 2APB potentiation in Parental and GemR AsPC-1 cells where “n = x,y” where “x” denotes the number of ROI in each condition and “y” denotes the number of dishes recorded. ∗∗∗∗*p* = 0.0011, ∗∗∗∗*p* < 0.0001 Data presented are mean ± SD. For statistical analysis, a one-sample *t* test was performed for *panels A* and *B*, while an unpaired student’s *t* test was used for *panels E* and *F* using GraphPad Prism software. Here, ∗*p* < 0.05; ∗∗*p* < 0.01; ∗∗∗*p* < 0.001 and ∗∗∗∗*p* < 0.0001.

### Orai3 drives gemcitabine resistance *in vitro*

To investigate if Orai3 overexpression contributes to acquired chemoresistance, we generated Orai3 stable knockdown cell line in the background of GemR CFPAC-1 and AsPC-1 cells. Orai3 knockdown in the stable line was validated by performing a Western blot. We observed a significant reduction in Orai3 expression in shOrai3 (shRNA specifically targeting Orai3) stable line compared to control shNT (non-targeting shRNA) line in GemR background in CFPAC-1 (Fig. 3*A*) and in AsPC-1 (Fig. 3*D*). To validate the functional role of Orai3 in gemcitabine-resistant cells, Fura-2 AM-based live cell Ca^2+^ imaging was performed in shNT GemR and shOrai3 GemR CFPAC-1 and AsPC-1 cells. A marked decrease in both Ca^2+^ entry and 2APB potentiation was observed upon Orai3 silencing in both CFPAC-1 GemR cells (Fig. 3, *B* and *C*) and AsPC-1 GemR cells (Fig. 3, *E* and *F*) compared to control. Collectively, this imaging data demonstrates that Orai3 constitutes a functional channel in CFPAC-1 GemR and AsPC-1 GemR cells. Subsequently, the role of Orai3 in modulating the diverse characteristics of GemR cells was investigated through a series of cell-based assays. We first investigated the proliferation ability of the four cell lines in the CFPAC-1 background: parental, GemR, shNT GemR, and shOrai3 GemR. We observed that the proliferation rate in GemR and shNT GemR was higher than parental and shOrai3 GemR cells. Interestingly, the increase in cell proliferation observed in GemR compared to parental cells was completely abrogated in shOrai3 GemR while it was comparable in shNT GemR (Fig. 3*G*). This suggests that GemR cells have higher proliferative capabilities compared to parental cells, and Orai3 knockdown drastically decreases the enhanced proliferative capabilities of GemR cells. Next, we analyzed cell cycle progression using propidium iodide staining to investigate the underlying cause of the differential proliferation rates observed under these conditions. Our analysis revealed that both parental and shOrai3 GemR cells exhibited arrest at the G1/S phase. In contrast, GemR and shNT GemR cells showed significant progression into the S-phase, as indicated by the distribution of cell populations across different cell cycle stages (Fig. 3*H*). The representative graphs of the cell cycle for all the conditions are shown (Fig. S3). Together, these findings demonstrate that Orai3 silencing significantly reduces the proliferation of GemR cells, primarily due to cell cycle arrest in the G1/S phase.

**Figure 3:**
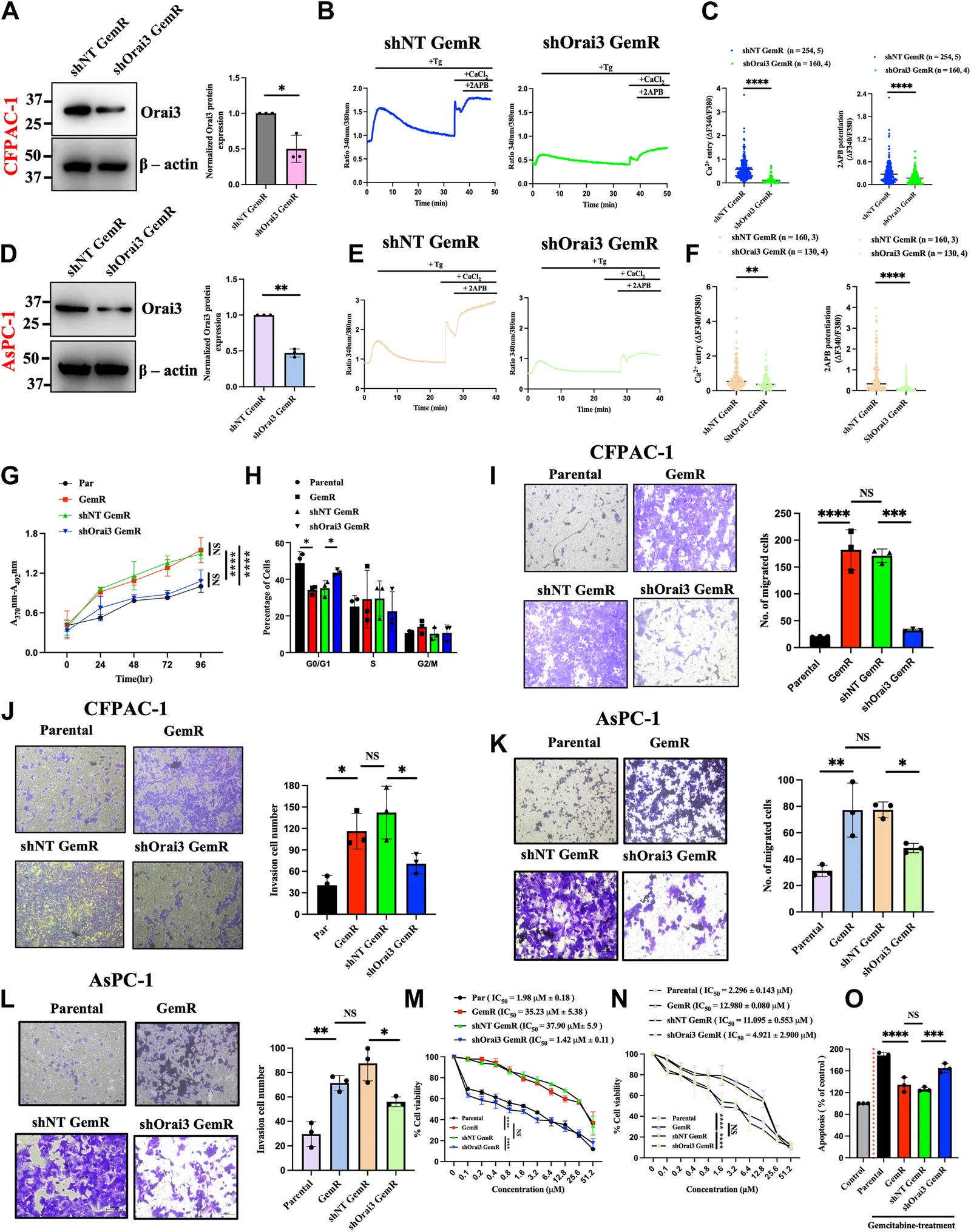
**Orai3 regulates gemcitabine resistance *in v*itro.***A*, representative Western blot and densitometry of Orai3 lentiviral stables generated in CFPAC-1 GemR cells at protein level (N = 3). ∗*p* = 0.0452. *B*, representative Ca2+ imaging traces of shNT and shOrai3 CFPAC-1 GemR cells where “n” denotes the number of cells in the trace. *C*, quantitation of Ca2+ entry and 2APB potentiation in shNT and shOrai3 CFPAC-1 GemR cells where “n = x,y” where “x” denotes the number of ROI in each condition and “y” denotes the number of dishes recorded. ∗∗∗∗*p* < 0.0001, ∗∗∗∗*p* < 0.0001. *D*, representative Western blot and densitometry of Orai3 lentiviral stables generated in AsPC-1 GemR cells at protein level (N = 3). ∗∗*p* = 0.0038. *E*, representative Ca2+ imaging traces of shNT and shOrai3 CFPAC-1 GemR cells, where “n” denotes the number of cells in the trace. *F*, Quantitation of Ca2+ entry and 2APB potentiation in shNT and shOrai3 AsPC-1 GemR cells, where “n = x,y” where “x” denotes the number of ROI in each condition and “y” denotes the number of dishes recorded. ∗∗*p* = 0.0011, ∗∗∗∗*p* < 0.0001. *G*, BrdU proliferation assay representing the proliferation rate upon silencing Orai3 at different time points (N = 3). ∗∗∗∗*p* < 0.0001, ∗∗∗∗*p* < 0.0001. *H*, quantitation of cell number in different cell cycle phases calculated for four conditions (N = 3). ∗*p* = 0.0195, ∗*p* = 0.0333. *I*, Boyden chamber transwell assay was performed to examine migration potential in parental, GemR, shNT, and shOrai3 CFPAC-1 GemR cells, and quantification of the number of cells migrated in these conditions. Images were captured using a Nikon bright field microscope at 10× (N = 3). ∗∗∗∗*p* < 0.0001, ∗∗∗*p* = 0.0001. *J*, Transwell assay for measuring invasion potential in parental, GemR, shNT, and shOrai3 CFPAC-1 GemR cells and quantification of the number of cells that invade the Matrigel coating on the transwell due to FBS gradient is shown graphically. Images were captured using a Nikon bright-field microscope at 10× (N = 3). ∗*p* = 0.0218, ∗*p* = 0.0292. *K*, Boyden chamber transwell assay was performed to examine migration potential in parental, GemR, shNT, and shOrai3 AsPC-1 GemR cells and quantification of the number of cells migrated in these conditions. Images were captured using a Nikon bright-field microscope at 10× (N = 3). ∗∗*p* = 0.004, ∗*p* = 0.049. *L*, Transwell assay for measuring invasion potential in parental, GemR, shNT, and shOrai3 AsPC-1 GemR cells and quantification of the number of cells that invade the Matrigel coating on the transwell due to FBS gradient is shown graphically. Images were captured using a Nikon bright-field microscope at 10× (N = 3). ∗∗*p* = 0.003, ∗*p* = 0.0157. *M*, MTT assay demonstrating the alteration in gemcitabine sensitivity following Orai3 silencing in GemR CFPAC-1 cells, as reflected by changes in IC50 values (N = 3). ∗∗∗∗*p* < 0.0001, ∗∗∗∗*p* < 0.0001. *N*, MTT assay demonstrating the alteration in gemcitabine sensitivity following Orai3 silencing in GemR AsPC-1 cells, as reflected by changes in IC50 values (N = 3). ∗∗∗∗*p* < 0.0001, ∗∗∗∗*p* < 0.0001. *O*, flow cytometric apoptosis analysis performed using Annexin V and DAPI for parental, GemR, shNT, and shOrai3 GemR CFPAC-1 cells alone or upon treatment with gemcitabine, and the quantification of apoptotic cells under each condition is presented. Apoptotic cell counts were determined by summing early and late apoptotic cells and normalizing the total to its respective control (N = 3). ∗∗∗∗*p* < 0.0001, ∗∗∗*p* = 0.0008. Data presented are mean ± SD. One-sample *t* test was performed for *panels A* and *D*, an unpaired student *t* test was performed for *panels C*, *F* and *H*, an ordinary one-way ANOVA followed by Tukey’s multiple comparisons test was done for *panels I*, *J*, *K*, *L*, and *O*. A two-way ANOVA followed by Tukey’s multiple comparisons test was performed for *panels G*, *M*, and *N* using GraphPad Prism software. Here, ∗*p* < 0.05; ∗∗*p* < 0.01; ∗∗∗*p* < 0.001 and ∗∗∗∗*p* < 0.0001.

Chemoresistance augments tumor cell migration and metastatic capabilities. Therefore, we studied the role of Orai3 in regulating the migration and invasion potential of GemR cells. We performed transwell assays to assess the migratory and invasion potential of parental, GemR, shNT GemR and shOrai3 GemR CFPAC-1 and AsPC-1 cells. Our data revealed that migration and invasion are highest in GemR and shNT GemR cells in both CFPAC-1 and AsPC-1. In contrast, parental and Orai3-silenced GemR cells exhibited significantly reduced migration (Fig. 3, *I* and *K*) and invasion (Fig. 3, *J* and *L*) in both CFPAC-1 and AsPC-1. This data demonstrates a positive correlation between Orai3 expression and migratory/invasion potential of GemR cells. The findings from *in vitro* functional assays using shRNA were further corroborated by transient silencing of Orai3 with an additional siRNA in CFPAC-1 GemR cells (Fig. S2), thereby confirming that Orai3 governs the diverse functional characteristics of GemR cells *in vitro*.

Cell migration is a highly polarized process that requires remodeling of the actin cytoskeleton to coordinate protrusion, adhesion, contraction, and retraction. To examine the role of Orai3 in regulating the actin cytoskeleton in GemR cells, we performed phalloidin staining in shNT GemR and shOrai3 GemR CFPAC-1 cells. Our data revealed a significant shift in the distribution of actin filaments across cells under these conditions (Fig. S4, *A* and *B*). Orai3 knockdown GemR cells showed actin distribution more at the center and less at the edges in contrast to shNT GemR cells, where the actin filaments are distributed more at the edges with a reduced presence towards the center. Also, analysis of our transcriptomic data showed decreased Rho GTPase levels upon silencing Orai3 in CFPAC-1 GemR cells, further confirming the pattern of actin distribution observed in these cells (Fig. S4*C*). Therefore, our data suggest that Orai3 regulates actin dynamics in GemR cells, thereby promoting their migration.

### Orai3 silencing sensitizes PC cells to gemcitabine by enhancing the rate of apoptosis

Next, we evaluated the role of Orai3 in protection against gemcitabine-induced cytotoxicity. We silenced Orai3 in GemR PC cells and assessed gemcitabine cytotoxicity at increasing doses using the MTT cell viability assay. Our findings showed that Orai3 knockdown in GemR cells restored gemcitabine sensitivity, as reflected by a decrease in IC_50_ values in CFPAC-1 GemR (Fig. 3*M*) and AsPC-1 GemR (Fig. 3*N*) cells. Interestingly, Orai3 silencing increases gemcitabine sensitivity, as observed in parental cells. The heightened chemosensitivity to gemcitabine observed in shOrai3 GemR cells could be due to increased apoptosis. To investigate this, we performed flow cytometry-based apoptosis assays. The four conditions of CFPAC-1 cells were treated with 5 μM gemcitabine for 72 h and stained with DAPI and annexin V-TRITC to distinguish apoptotic and necrotic cells. Our data show a significant increase in apoptosis in parental and Orai3-silenced cells compared to GemR and shNT GemR cells (Figs. S3*B* and 3*O*). Collectively, our data suggest that Orai3 is critical for providing a survival advantage to GemR cells by preventing apoptosis.

### Orai3 enhances the stemness of gemcitabine-resistant cells

Cancer stemness is a critical determinant of chemoresistance, contributing to recurrence and drug resistance in pancreatic cancer (26, 27). To investigate Orai3’s contribution to the stemness of gemcitabine-resistant cells, we generated lentiviral-based stable knockdown of Orai3 in GemR cells. The stemness of these cells was first confirmed using 3D spheroid formation assays. The spheroid size was used as a parameter to measure differences among the four conditions. Interestingly, the bright-field image showed a well-formed, significantly larger spheroid in GemR cells than in parental cells, while Orai3-silenced cells generated spheroids smaller than those from shNT GemR cells in both CFPAC-1 (Fig. 4*A*) and AsPC-1 (Fig. 4*B*) cells. The GFP spheroid images of shNT and shOrai3 GemR are also shown. A seminal study identified a subset of pancreatic cancer stem cells *in vivo*, which were CD44+ CD24+ ESA+, and harbored greater tumorigenic potential (28). These triple-positive cells are enriched in gemcitabine-resistant pancreatic cancer cells (16). Further, B-cell-specific Moloney murine leukemia virus insertion site 1 (BMI1) plays a crucial role in cancer pathogenesis. Pancreatic cancer stem cells isolated from both tumor spheres and xenografts based on the expression of CD44, CD24, and ESA were shown to have significantly higher expression of BMI1 (29). Therefore, we evaluated the expression of these key stemness players and observed a significant reduction in their expression upon silencing Orai3 (Fig. 4*C*). Furthermore, literature suggests that aldehyde dehydrogenase (ALDH), a critical enzyme in regulating the retinoic acid pathway, and its isoforms are overexpressed in cancer stem cells (30, 31, 32, 33). In pancreatic cancer, ALDH1A1 knockdown significantly reduced gemcitabine resistance, highlighting ALDH1A1's important role in acquired resistance to gemcitabine (34). Hence, we examined ALDH1A1 expression and observed that it was significantly upregulated in CFPAC-1 GemR (Fig. 4*D*) and AsPC-1 GemR (Fig. 4*E*) cells compared to the parental cells, and upon Orai3 silencing in GemR cells, ALDH1A1 expression is significantly reduced in both cell lines compared to their respective controls. Taken together, our data demonstrate that Orai3 regulates cancer stemness properties and the expression of key drivers of cancer stemness in gemcitabine resistant pancreatic cancer cells.

**Figure 4:**
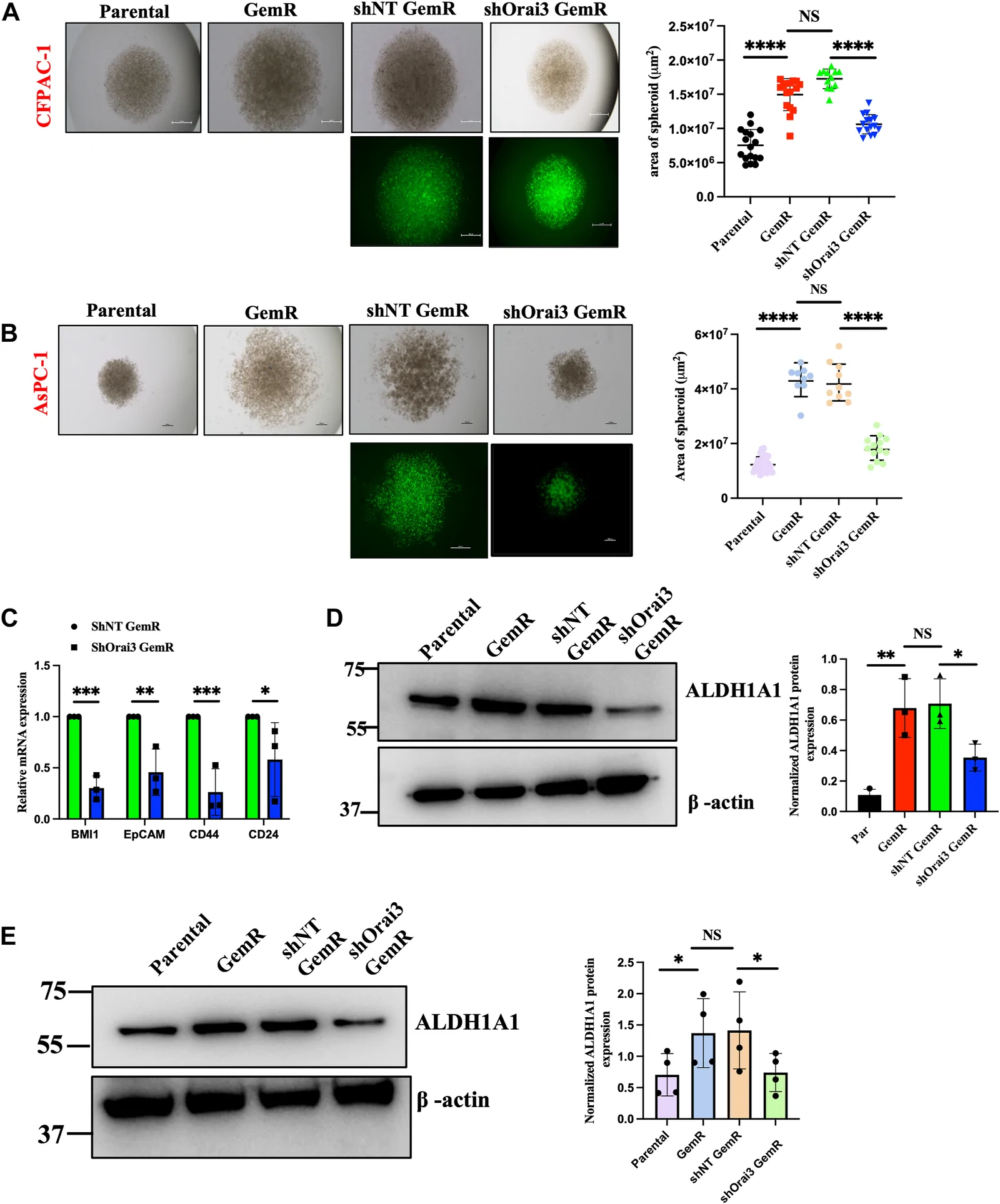
**Orai3 regulates the stemness of gemcitabine resistant cells.***A*, spheroid formation assay in CFPAC-1 GemR cells was conducted using the hanging drop method. Each spheroid was defined as a region of interest (ROI), and its area was measured using Nikon software. The quantified area is represented in the graph (N = 3). ∗∗∗∗*p* < 0.0001, ∗∗∗∗*p* < 0.0001. *B*, spheroid formation assay in AsPC-1 GemR cells was conducted using the hanging drop method. Each spheroid was defined as a region of interest (ROI), and its area was measured using Nikon software. The quantified area is represented in the graph (N = 3). ∗∗∗∗*p* < 0.0001, ∗∗∗∗*p* < 0.0001. *C*, qRT-PCR analysis demonstrates various stemness markers' expression following Orai3 silencing in GemR CFPAC-1 cells (N = 3). using Nikon software. The quantified area is represented in the graph (N = 3). ∗∗∗*p =* 0.0007, ∗∗*p =* 0.0066, ∗∗∗*p =* 0.0004, ∗*p =* 0.0398. *D*, representative Western blot showing the expression of ALDH1A1 across different conditions in CFPAC-1 GemR cells, and densitometric analysis is done using ImageJ and presented graphically (N = 3). ∗∗*p =* 0.0037, ∗*p =* 0.0489. *E*, representative Western blot showing the expression of ALDH1A1 across different conditions in AsPC-1 GemR cells, and densitometric analysis is done using ImageJ and presented graphically (N = 3). ∗*p =* 0.0126, ∗*p =* 0.0117 The data presented is mean ± SD. An ordinary one-way ANOVA followed by Tukey’s multiple comparisons test was performed for *panels A*, *B*, *D*, and *E*, and a one-sample *t* test was performed for *panel C* using GraphPad Prism software for statistical analysis. Here, ∗*p* < 0.05; ∗∗*p* < 0.01; ∗∗∗*p* < 0.001 and ∗∗∗∗*p* < 0.0001.

### Orai3 enhances metastatic potential of GemR cells *in vivo*

To study the role of Orai3 in regulating the metastatic potential of GemR cells *in vivo*, we used zebrafish (*D. rerio*) as a model system. The initiation of metastasis involves the migration of tumor cells from the primary tumor to other parts of the body, where they form secondary tumors. To replicate this process, we first optimized and generated CFPAC-1 xenografts in zebrafish. The four types of CFPAC-1 cell line, *that is*, parental, GemR, shNT GemR, and shOrai3 GemR, were labeled with CM-DiI (a red colored dye) and injected into the perivitelline space (PVS) of zebrafish embryos at 2 days post-fertilization (dpf) as shown in the schematic diagram (Fig. 5*A*). Subsequently, to determine the metastatic potential, each xenograft was analyzed at 4 days post-injection (dpi) to assess for micro-metastasis in the caudal hematopoietic tissue (CHT). The representative images of Parental, GemR, shNT, and shOrai3 GemR xenografts with metastasis in CHT are shown (Fig. 5*B*), and the percentage of metastasis is calculated using the following formula.

$$\text{Met}\;\%=\frac{\text{no}.\;\text{of}\;\text{live}\;\text{xenografts}\;\left(4\text{dpi}\right)\;\text{with}\;\text{micro}-\text{metastasis}\;\text{in}\;\text{CHT}\;X100}{\text{Total}\;\text{no}.\;\text{of}\;\text{live}\;\text{xenografts}\;\text{at}\;4\text{dpi}}$$

**Figure 5:**
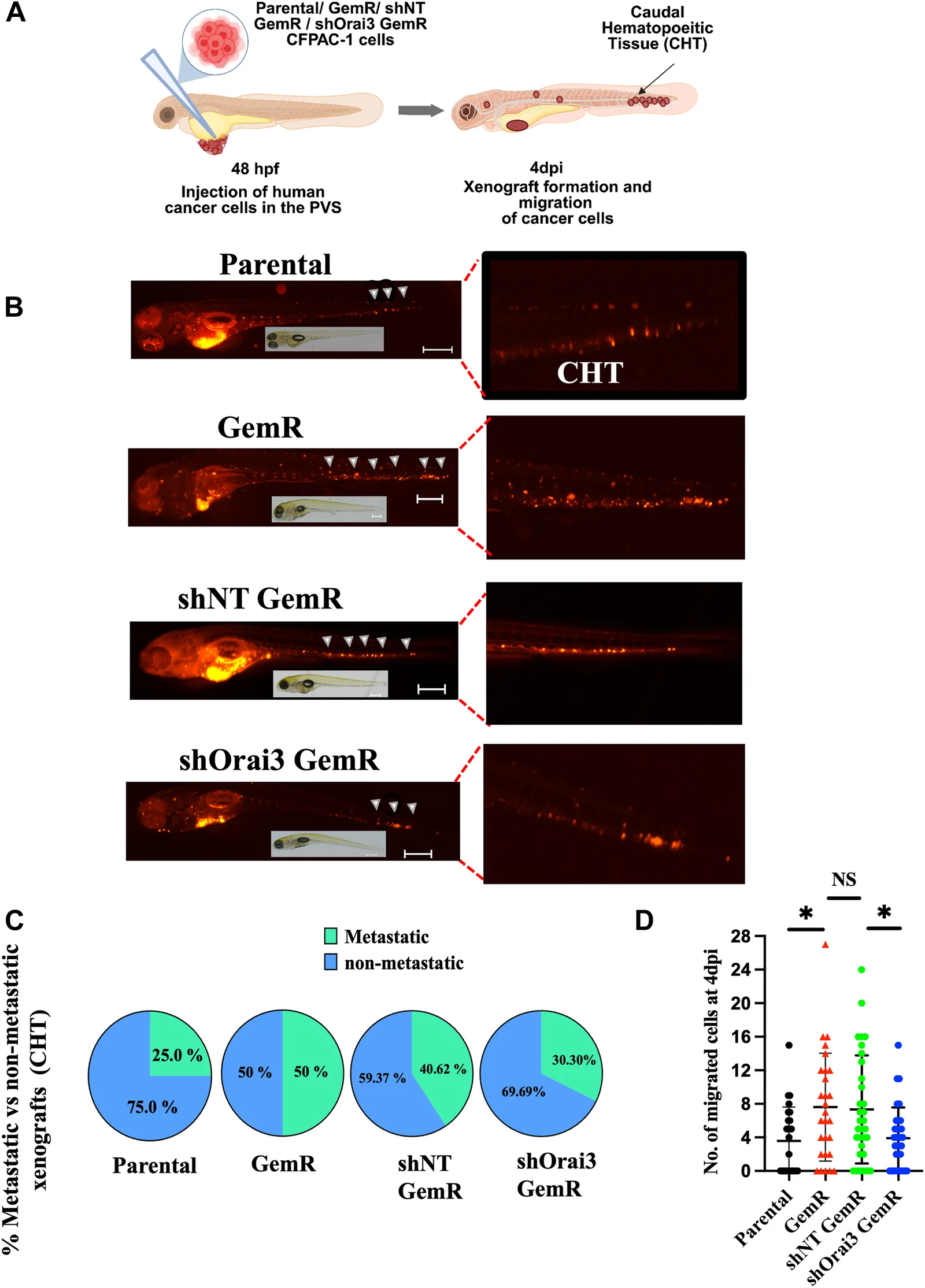
**Orai3 regulation of GemR metastatic potential *in vivo*.***A*, schematic depicting cell microinjection into 2dpf zebrafish larvae, xenograft formation, and cell migration to the CHT at 4dpi. *B*, representative zebrafish xenograft images showing migration of labeled cells injected in PVS at 2dpf to CHT at 4dpi. *C*, the percentages of metastatic *versus* non-metastatic xenografts in different conditions are shown in the pie chart. Parental (n = 28), GemR (n = 28), shNT GemR (n = 32), and shOrai3 GemR (n = 33). *D*, quantitation of the total number of cells migrated to different locations in xenografts is shown graphically. ∗*p =* 0.031, ∗*p =* 0.0497 Data presented are mean ± SD. An ordinary one-way ANOVA followed by Tukey’s multiple comparisons test was performed for *panel D* using GraphPad Prism software. Here, ∗*p* < 0.05; ∗∗*p* < 0.01; ∗∗∗*p* < 0.001 and ∗∗∗∗*p* < 0.0001. The ‘n’ denotes the total number of xenografts analyzed for quantitation.

Total no. of live xenografts at 4dpi.

Using this formula, we calculated that 50.0% and 40.62% xenografts in GemR and shNT GemR cells exhibited metastasis to the CHT, while Orai3 silencing reduced this to 30.30%, which is comparable to parental cell xenografts that showed 25.0% metastatic xenografts (Fig. 5*C*). Additionally, we quantified the number of cells that metastasize to different sites in the zebrafish body at 4dpi using ImageJ software. As expected, our data revealed that GemR cells have greater metastatic potential than parental cells (Fig. 5*D*). Importantly, we observed a significant reduction in metastatic cells in shOrai3 GemR as compared to shNT GemR cells (Fig. 5*D*). Taken together, our findings highlight a crucial role for Orai3 in driving the metastasis of GemR cells *in vivo*.

### Unbiased transcriptomics reveals that SLIT3 functions downstream of Orai3

Our *in vitro* and *in vivo* data demonstrate that Orai3 plays a pivotal role in sustaining gemcitabine resistance in pancreatic cancer cells. Next, we performed bulk RNA seq to unveil the molecular mechanisms that contribute to acquired gemcitabine resistance downstream of Orai3. RNA seq was performed on all four conditions: parental, GemR, shNT GemR and shOrai3 GemR CFPAC-1 cells (Fig. 6*A*). The fold change was calculated for test conditions GemR and shOrai3 GemR with respect to (w.r.t.) their respective control, *i.e.*, parental and shNT GemR. Genes showing a log2 fold change of +2 w.r.t. control were considered upregulated genes, while a cut-off log2 fold change of less than −2 was set to filter out downregulated genes. The differentially expressed genes (DEGs) were screened using |logFC| > 2 and *p* ≤ 0.05. The differentially expressed genes for GemR *versus* parental (Fig. S5*A*) and shOrai3 GemR *versus* shNT GemR (Fig. S5*B*) groups are shown in the volcano plots. We also examined the biological, cellular, and molecular pathways associated with gemcitabine resistance through GO pathway enrichment analysis in the group GemR *versus* parental (Fig. S5, *C* and *D*) and shOrai3 GemR *versus* shNT GemR (Fig. S5, *E* and *F*). To identify the genes that are common but differentially regulated in parental *versus* GemR and shNT *versus* shOrai3 GemR groups, we looked for common genes that were upregulated in the GemR cells *versus* parental but downregulated in shOrai3 GemR in comparison to shNT GemR. This analysis identified 12 genes with differential expression. Since the Orai3-mediated effect is most likely due to its Ca^2+^ signaling function, we further overlapped these 12 genes with the Ca^2+^-associated genes database (35). This database comprises all the genes implicated in Ca^2+^ signaling and linked to human disorders. This robust analysis revealed only one gene, SLIT3, that was common among the three groups (Fig. 6*B*). SLIT3 belongs to a family of secreted proteins that bind to the roundabout (ROBO) family of transmembrane proteins, serving as an axonal guidance cue during nervous system development. (36). Further, recent studies have shown that SLIT3 is crucial for cancer initiation and progression (37).

**Figure 6:**
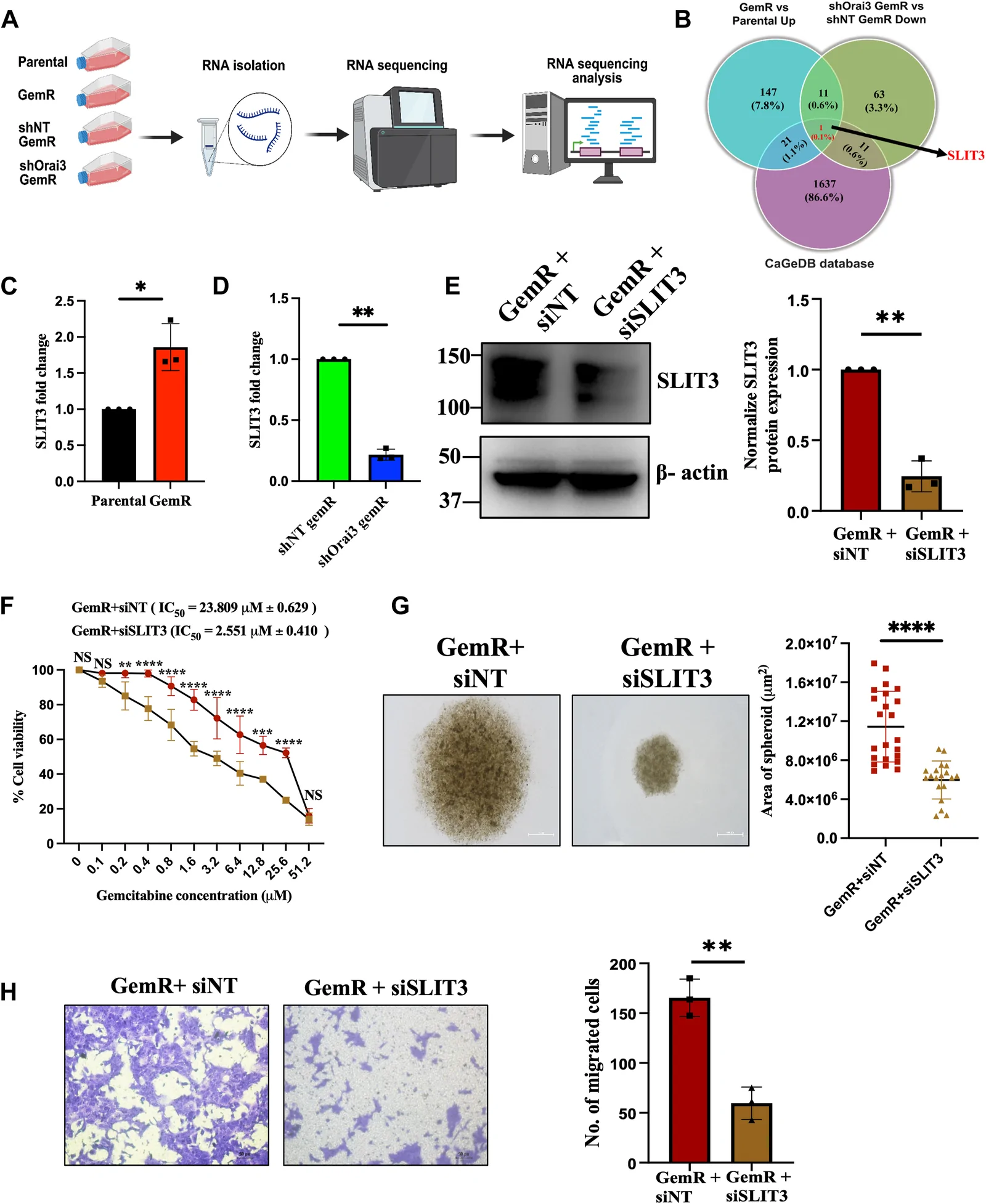
**SLIT3 positively regulates gemcitabine resistance.***A*, schematic diagram showing steps for RNA-sequencing workflow. *B*, Venn diagram representing common differentially expressed genes in GemR *versus* Parental up overlapped and shOrai3 GemR *versus* shNT GemR down overlapped with Ca^2+^-regulated genes. *C*, validation of SLIT3 mRNA expression in parental *versus* GemR (N = 3). ∗*p =* 0.0445. *D*, validation of SLIT3 mRNA expression in shNT *versus* shOrai3 GemR CFPAC-1 cells (N = 3). ∗∗*p =* 0.0011. *E*, representative Western blot confirming siRNA-based silencing of SLIT3 in GemR cells. Densitometric analysis of SLIT3 knockdown using ImageJ is shown graphically (N = 3). ∗∗*p =* 0.0069. *F*, MTT assay shows the change in the chemosensitivity of GemR cells upon silencing SLIT3 (N = 3). ∗∗*p =* 0.0068, ∗∗∗*p* = 0.0001, ∗∗∗∗*p* < 0.0001. *G*, Spheroid formation assay depicting a spheroid's size change on silencing SLIT3 in GemR cells. Each spheroid was defined as a region of interest (ROI), and its area was measured using Nikon software. The quantified area of spheroids is represented in the graph (N = 3). ∗∗∗∗*p* < 0.0001. *H*, Boyden chamber transwell assay for studying the migration potential of GemR cells upon silencing SLIT3. Graph showing a quantitative analysis of migration potential in SLIT3-knockdown cells (N = 3). ∗∗*p* = 0.0018 Data presented are mean ± SD. A one-sample *t* test was performed for *panels C*, *D*, and *E*, a two-way ANOVA followed by Tukey’s multiple comparisons test was performed for *panel F*, and an unpaired Student *t* test for *panels G* and *H* using GraphPad Prism software. Here, ∗*p* < 0.05; ∗∗*p* < 0.01; ∗∗∗*p* < 0.001 and ∗∗∗∗*p* < 0.0001.

Interestingly, whole-genome studies on samples of patient with PC have revealed mutations in the SLIT and ROBO genes (38, 39, 40). These studies have highlighted a strong correlation between the dysregulated SLIT/ROBO signaling and early stages of pancreatic neoplastic transformation (39). However, SLIT3’s role in pancreatic cancer chemoresistance remains unappreciated. Therefore, based on transcriptomic data analysis and a literature survey, we focused on understanding the role of SLIT3 in Orai3-induced chemoresistance. We first examined SLIT3 expression in the samples used for RNA sequencing to validate our transcriptomic results. As observed in transcriptomics, SLIT3 expression is higher in GemR cells in comparison to parental cells (Fig. 6*C*), while it decreases in the Orai3-silenced GemR cells as compared to shNT control GemR cells (Fig. 6*D*). To understand the role of SLIT3 in gemcitabine resistance, we started by silencing SLIT3 in GemR cells using siRNA. As shown in our Western blotting data, there is a robust reduction in SLIT3 protein levels upon transfection with SLIT3-targeting siRNA (Fig. 6*E*). Subsequently, siSLIT3-transfected cells were used to examine the role of SLIT3 in chemoresistance. First, we performed MTT assays to examine the role of SLIT3 in chemosensitivity. We observed that SLIT3 knockdown in these cells increases sensitivity to gemcitabine, as we see a robust (10-fold) decrease in IC_50_ values of gemcitabine (Fig. 6*F*). To examine the effect of SLIT3 silencing on stemness, we performed 3-D spheroid assays that revealed a drastic reduction in the spheroid area (Fig. 6*G*). Furthermore, the transwell migration studies show that SLIT3 knockdown in GemR cells results in a significant decrease in migration potential compared to control (Fig. 6*H*). Altogether, our results uncover that downstream of Orai3, SLIT3 expression is augmented in GemR cells. Further, SLIT3 plays an important role in enhancing chemoresistance, migration and stemness of GemR cells thereby driving gemcitabine resistance.

### SLIT3 drives gemcitabine resistance downstream of Orai3

We observed that SLIT3 expression is increased in GemR cells, and it is decreased in Orai3-silenced GemR cells. Further, we found that SLIT3 contributes to GemR in pancreatic cancer cells. Next, to examine whether SLIT3 contributes to gemcitabine resistance downstream of Orai3, we performed overexpression of SLIT3 in Orai3-silenced GemR cells. SLIT3 was transfected into Orai3-silenced GemR cells, resulting in significant overexpression of SLIT3 (Fig. 7*A*). We subsequently examined chemosensitivity of these cells by performing MTT assays at different gemcitabine concentrations. These experiments demonstrated a substantial increase in chemoresistance in SLIT3 overexpressing Orai3-silenced GemR cells compared to the Orai3-silenced GemR cells (Fig. 7*B*). Further, 3-D spheroid formation assays showed enhanced stemness properties (Fig. 7*C*) and transwell assays uncovered higher migration (Fig. 7*D*) & invasion (Fig. 7*E*) potential in SLIT3 complemented cells in comparison to Orai3 silenced GemR cells. Collectively, our data reveal that overexpression of SLIT3 in Orai3-silenced GemR cells restores chemoresistance, migration, invasion and stemness. Hence, these data confirms that SLIT3 works downstream of Orai3 to drive gemcitabine resistance.

**Figure 7:**
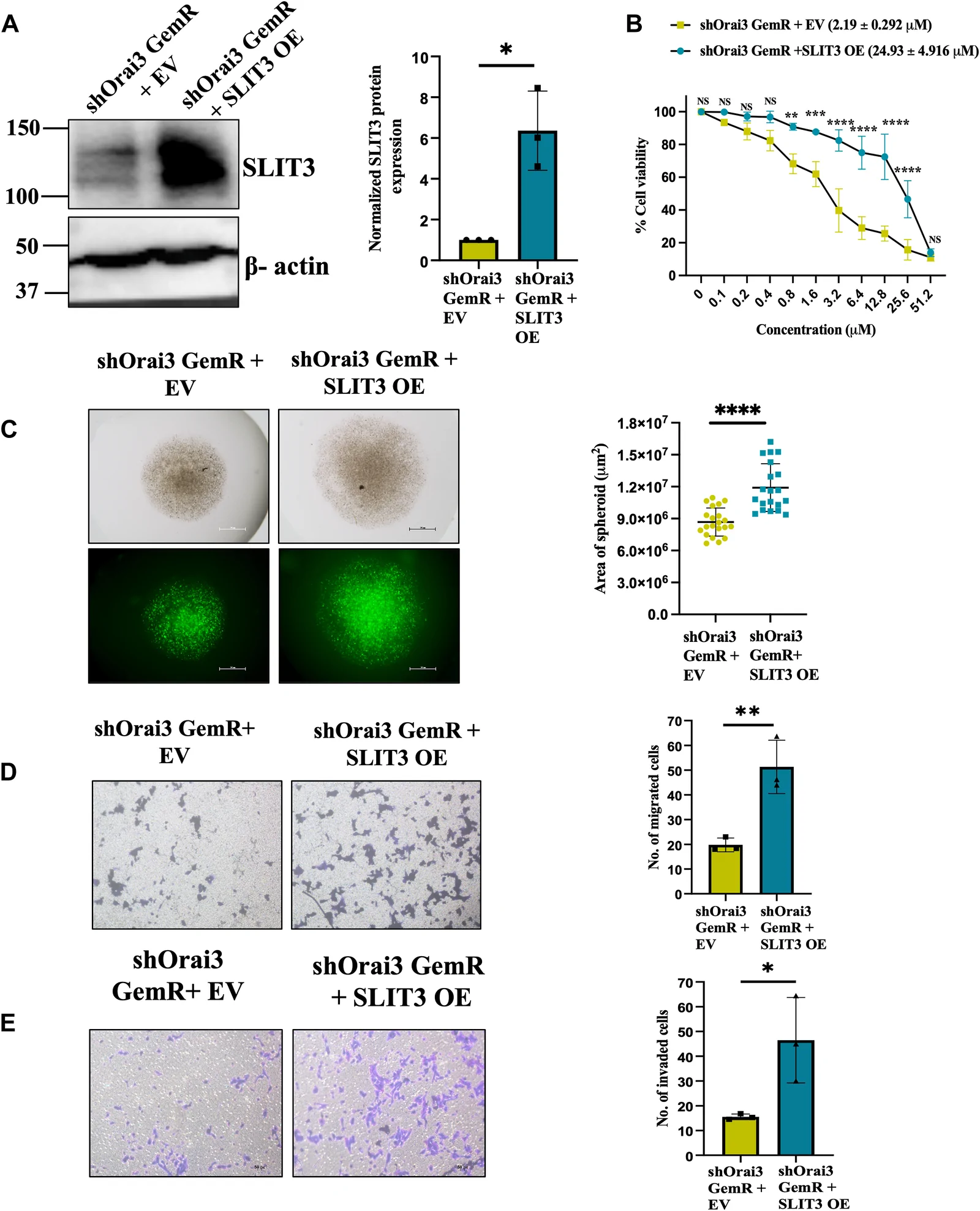
**SLIT3 functions downstream of Orai3 to regulate gemcitabine resistance.***A*, Representative Western blot confirming overexpression of SLIT3 in Orai3-silenced GemR cells. Densitometric blot analysis using ImageJ is shown graphically (N = 3). ∗*p* = 0.0411. *B*, MTT assay shows the change in chemosensitivity of shOrai3 GemR cells following overexpression of SLIT3 compared to empty vector control (N = 3). ∗∗*p =* 0.0013, ∗∗∗*p =* 0.0002, ∗∗∗∗*p* < 0.0001. *C*, spheroid formation assay depicting the shape change of the spheroid upon overexpression of SLIT3 in shOrai3 GemR cells. Each spheroid was defined as a region of interest (ROI), and its area was measured using Nikon software. The quantified area of spheroids is represented in the graph (N = 3). ∗∗∗∗*p* < 0.0001. *D*, Boyden chamber transwell assay showing migration potential of shOrai3 GemR cells upon overexpression of SLIT3.Quantitation of migration is shown graphically (N = 3). ∗∗*p =* 0.0080. *E*, Boyden chamber transwell assay with Matrigel coating showing invasion ability of shOrai3 GemR cells upon overexpression of SLIT3.Quantitation of invasion is shown graphically (N = 3). ∗*p =* 0.0361 Data presented are mean ± SD. A one-sample *t* test was performed for *panel A*. A two-way ANOVA followed by Šídák's multiple comparisons test was done for *panel B*, and an unpaired Student's *t* test for *panels C*, *D*, and *E* using GraphPad Prism software. Here, ∗*p* < 0.05; ∗∗*p* < 0.01; ∗∗∗*p* < 0.001 and ∗∗∗∗*p* < 0.0001.

### SLIT3 expression is transcriptionally regulated by NFATc1

Our transcriptomics data identified SLIT3 as a differentially regulated gene downstream of Orai3 in GemR cells. To delineate the molecular mechanism driving SLIT3’s transcriptional regulation by Orai3, we investigated potential role of the Nuclear Factor of Activated T cells (NFAT) transcription factors. The activation of NFATc1-c4 requires a sustained Ca^2+^ signal, which is achieved by the opening of Orai channels at the plasma membrane (41, 42, 43). We performed extensive bioinformatic analysis to examine the potential binding sites of NFATs on SLIT3 promoter using two independent tools, namely, the Eukaryotic Promoter Database (EPD) search motif tool (https://epd.expasy.org/epd/) and PSCAN (Tang *et al.*, 2020). The databases revealed five potential binding sites for NFATc2 and four binding sites for NFATc1 and NFATc3 on the SLIT3 promoter after applying the stringent *p*-value cutoff of 0.001 (Fig. 8*A*), while no binding site for NFATc4 was predicted by these tools. PSCAN was used to identify the sequence of putative TF-binding sites in the SLIT3 promoter (Fig. 8*B*) using the JASPAR Core 2020 non-redundant transcription factor position weight matrix database. These two tools, each using a different algorithm, identified putative binding sites and a putative consensus sequence present on the SLIT3 promoter for different NFATs. Thus, our robust bioinformatic analysis suggests that NFATs can potentially regulate SLIT3 transcription downstream of Orai3.

**Figure 8:**
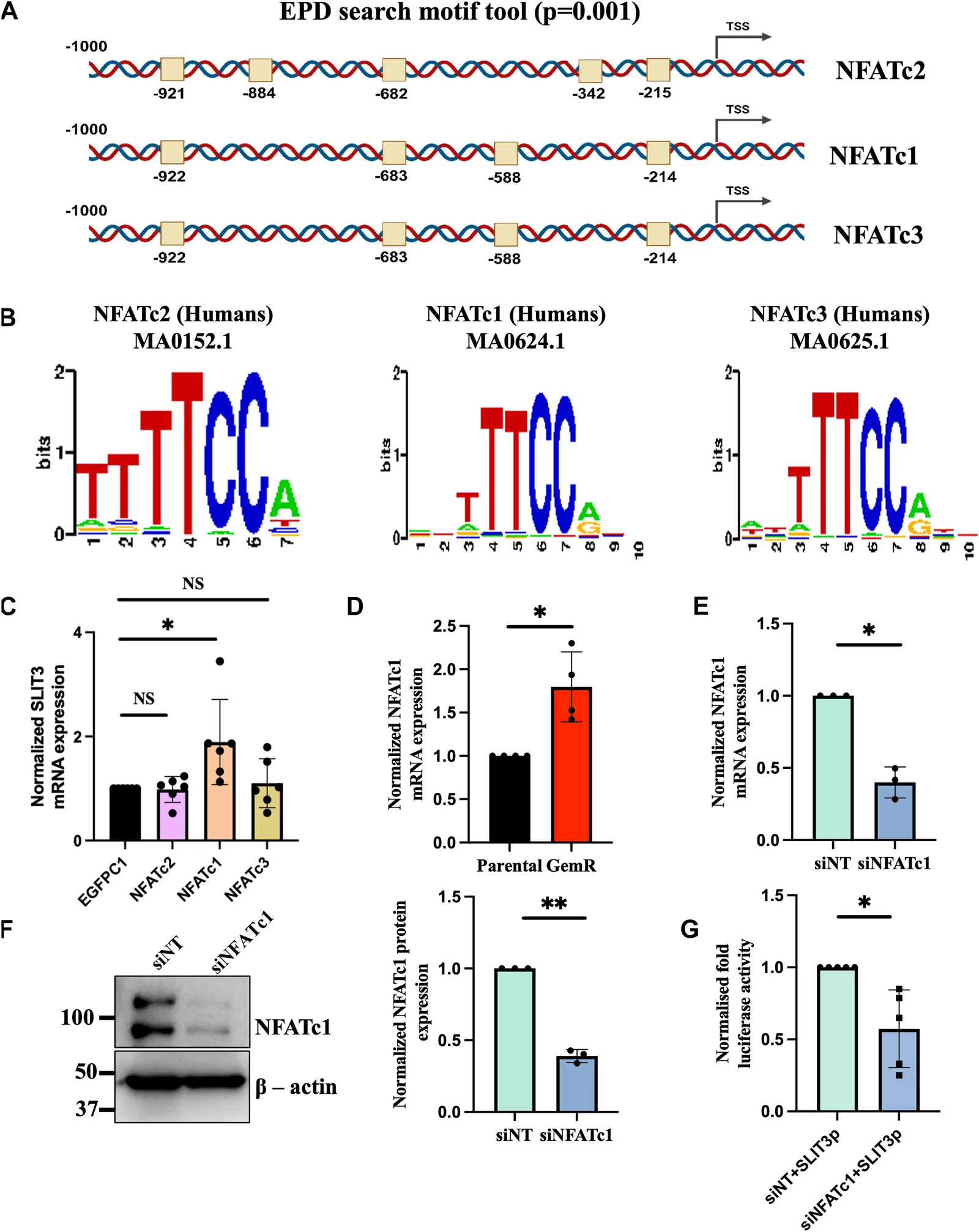
**SLIT3 is transcriptionally regulated by NFATc1 in GemR cells.***A*, identification of putative binding sites on SLIT3 promoter using the EPD tool at a *p*-value cut-off of 0.001. *B*, A position weight matrix of human NFATc2/c1/c3 consensus binding sequence on SLIT3 promoter. *C*, SLIT3 mRNA expression following NFATc2/c1/c3 overexpression in the Parental CFPAC-1 cell line (N = 5). ∗*p* = 0.0228 *D*, mRNA expression of NFATc1 in Parental *versus* GemR CFPAC-1 cell line (N = 4). ∗*p* = 0.0291. *E*, knockdown validation of NFATc1 in CFPAC-1 GemR cells using qPCR (N = 3). ∗*p* = 0.0105. *F*, knockdown validation of NFATc1 in CFPAC-1 GemR cells using Western blot (N = 3). ∗∗*p* = 0.0019. *G*, normalized luciferase activity of SLIT3 promoter upon NFATc1 silencing in CFPAC-1 GemR cells (N = 5). ∗*p* = 0.0240 Data presented are mean ± SD. An ordinary one-way ANOVA was performed for *panel C* and a one-sample *t* test for *panels D*, *E*, *F*, and *G* using GraphPad Prism software. Here, ∗*p* < 0.05; ∗∗*p* < 0.01; ∗∗∗ ∗∗*p* < 0.01; ∗∗∗*p* < 0.001 and ∗∗∗∗*p* < 0.0001.

To identify which NFAT isoform regulates SLIT3 expression, we performed overexpression studies in the wild-type CFPAC-1 cell line, which has low SLIT3 expression. We observed that NFATc1 overexpression markedly increased SLIT3 mRNA levels compared to the empty vector, while the expression of SLIT3 was unchanged upon NFATc2 or NFATc3 overexpression (Fig. 8*C*). We further performed luciferase assays to assess the SLIT3 promoter activity in response to NFATc1 overexpression. Since NFATc1 expression is higher in CFPAC-1 GemR cells (Fig. 8*D*), we silenced NFATc1 in them and confirmed the knockdown at the RNA (Fig. 8*E*) and protein levels (Fig. 8*F*). Then, we overexpressed the SLIT3 promoter encompassing all four predicted SLIT3 binding sites cloned into the dual luciferase reporter vector in siNT and siNFATc1 cells. After 48 h of transfection, we performed luciferase assays to measure promoter activity. The results revealed a marked decrease in SLIT3 promoter activity following NFATc1 silencing (Fig. 8*G*), strongly suggesting that NFATc1 positively regulates SLIT3 transcription and is a key driver of its activation in GemR cells. Interestingly, upon Orai3 silencing in CFPAC-1 GemR cells, we observed NFATc1 downregulation, suggesting that Orai3-mediated activation of NFATc1 subsequently drives the transcriptional upregulation of SLIT3 expression in GemR cells (Fig. S6*A*). These findings further support our bioinformatic analysis and establish a functional role of NFATc1 in regulating SLIT3 transcription.

In summary, we demonstrate that Orai3 plays a crucial role in the acquired resistance to gemcitabine in PC. We performed a variety of *in vitro* assays and *in vivo* studies to demonstrate that Orai3 silencing enhances gemcitabine sensitivity in the resistant cells. Further, we performed unbiased transcriptomics analysis and identified that SLIT3 drives gemcitabine resistance downstream of Orai3. Moreover, we delineated that NFATc1 transcription factor connects Orai3 to SLIT3 regulation. In future, strategies for targeting Orai3 in combination with gemcitabine may pave the path for a more efficient chemotherapy regime against pancreatic cancer.

## Discussion

Pancreatic Cancer (PC) is one of the deadliest cancers and is associated with an extremely poor prognosis. Gemcitabine is one of the key chemotherapeutic drugs used for the clinical management of PC. However, acquired resistance to gemcitabine remains a significant clinical challenge, significantly limiting the efficacy of gemcitabine. With the limited effective second-line treatment options available, there is a critical need to develop novel therapeutic strategies that overcome gemcitabine resistance.

Ca^2+^ signaling is emerging as a key driving force in mediating the cytotoxic effects of chemotherapy as numerous chemotherapeutic agents trigger an extensive rewiring of cytosolic Ca^2+^ signaling in cancer cells (21). This phenomenon is primarily attributed to the dysregulated expression or activity of various plasma membrane Ca^2+^ ion channels (14, 44). In non-excitable cells, Ca^2+^ entry is primarily driven by a process known as Store-Operated Ca^2+^ Entry (SOCE), which is mediated by the Orai family of channels, specifically Orai1, Orai2, and Orai3 (13). Evolving evidence indicates that enhanced SOCE plays a crucial role in the development of chemoresistance. A recent study showed co-amplification of RRM1 and STIM1 in gemcitabine-resistant pancreatic cancer cells. The heightened expression of STIM1 correlates with elevated store-operated Ca^2+^ entry (SOCE) and dysregulated endoplasmic reticulum (ER) stress, resulting in enhanced NFATc2 activity and epigenetic reprogramming (10). Another intriguing study developed a gemcitabine-resistant pancreatic cancer cell line and used high-throughput omics platforms to identify calmodulin as a key driver of gemcitabine resistance. Inhibiting calmodulin with a Ca^2+^ channel blocker, amlodipine restored gemcitabine sensitivity *in vitro* and *in vivo* (45). Collectively, these studies highlight that augmented Ca^2+^ influx contributes to gemcitabine resistance, but the identity of the Ca^2+^ channel and downstream molecular mechanism remains elusive.

Orai3 encodes a SOCE channel in a subset of breast cancer cells and drives breast tumorigenesis (24, 46, 47). Further, our previous study demonstrated that Orai3 functions as a SOCE channel in PC and is essential for PC progression & metastasis (15). However, the functional relevance of Orai3 in acquired gemcitabine resistance remains unappreciated. Here, we report a novel role of Orai3 in acquired gemcitabine resistance. Using two different pancreatic cancer cell lines, we show that gemcitabine-based chemotherapy selectively increases Orai3 and STIM1 expression. In contrast, the expression levels of Orai1, Orai2, and STIM2 are downregulated or remain unaffected in both CFPAC-1 and AsPC-1 GemR cell lines.

Another intriguing observation was the increase in Ca^2+^ entry in GemR cells, highlighting gemcitabine's influence on cytosolic Ca^2+^ levels. The gemcitabine resistance augments SOCE in both CFPAC-1 GemR (Fig. 2*C*) and AsPC-1 GemR cells (Fig. 2*F*). Furthermore, the enhanced 2APB-mediated potentiation of Ca^2+^ entry in GemR cells confirms the functional role of Orai3 in both CFPAC-1 GemR (Fig. 2*C*) and AsPC-1 GemR cells (Fig. 2*F*). These data suggest that gemcitabine resistance results in elevation of Ca^2+^ entry that may impact drug sensitivity and may induce resistance mechanisms. Here, we reason that cancer cells may upregulate SOCE during chemotherapy as a survival mechanism. This adaptive response might help cancer cells to maintain Ca^2+^ homeostasis and activate pro-survival signals to resist chemotherapy-induced cell death. Therefore, targeting SOCE proteins under these conditions could disrupt the protective mechanism, making cancerous cells more vulnerable to chemotherapy. Indeed, our data clearly demonstrate that Orai3 plays a crucial role in acquired chemoresistance, and silencing Orai3 makes cells more susceptible to gemcitabine (Fig. 3, *M* and *N*). This suggests that targeting Orai3 could be a therapeutic strategy to enhance gemcitabine efficacy by sensitizing resistant cells to therapy.

The role of Orai proteins in the induction of cancer stem cell (CSC) phenotype has been highlighted in various cancers such as glioblastoma (48, 49) and lung cancer (50, 51, 52). The CSC properties include self-renewal ability, tumor spheroid formation, drug resistance, enhanced migratory potential, elevated expression of CSC markers (53, 54). Gemcitabine resistance can augment the CSC subpopulation through multiple pathways, including Nox/ROS/NF-κB/STAT3 and hypoxia-induced Akt/Notch1 signaling (27, 55). In our study, we demonstrate that Orai3 silencing in GemR cells reduces the expression of stemness markers CD44, CD24, EPCAM, BMI1, and ALDH1A1 (Fig. 4, *C*–*E*). Further, Orai3 plays a critical role in tumor spheroid formation (Fig. 4, *A* and *B*) and contributes to enhanced metastasis of GemR cells (Fig. 5). Collectively, our data reveal that Orai3 is crucial for regulating the stemness of GemR PC cells.

Cancer cell migration is a crucial step in metastasis and is regulated by changes in the structure and dynamics of the actin cytoskeleton. It is reported that spatiotemporal variations in intracellular Ca^2+^ levels have a key role in forming new protrusions at the leading edge and disassembling mature adhesions at the rear (27). Our study showed that silencing Orai3 significantly reduced the migration and invasion potential of CFPAC-1 GemR (Fig. 3, *I* and *J*) and AsPC-1 GemR cells (Fig. 3, *K* and *L*). To examine the underlying mechanism, we analyzed the distribution of actin filaments across the cell by staining them with phalloidin. Interestingly, we observed that in Orai3-silenced GemR cells, actin filaments were distributed more towards the nucleus and less towards the cell periphery than in control cells (Fig. S4, *A* and *B*). Recently, a connection between actin remodeling and chemoresistance was reported, showing that RhoJ facilitates EMT-associated resistance to chemotherapy (56). Rho GTPases are a family of small G-proteins that control tumor initiation and progression by regulating cell migration, proliferation, apoptosis and by influencing metabolism, senescence and cancer cell stemness (57). Analysis of our transcriptomic data showed downregulation of Rho GTPases in Orai3-silenced GemR cells compared with controls (Fig. S4*C*). This suggests that Orai3 regulates actin dynamics in GemR cells, thereby enhancing their migratory and metastatic potential.

For unbiased identification of the molecular mechanism working downstream of Orai3 to drive gemcitabine resistance, we performed RNA-seq analysis on parental, GemR, shNT and shOrai3 GemR cells. Our transcriptomics analysis identified SLIT3 (Fig. 6*B*), which acts downstream of Orai3 to induce gemcitabine resistance. SLITs are a class of secretory glycoprotein that binds to the roundabout guidance receptor (ROBO) family of transmembrane proteins, serving as an axonal guidance cue in developing the nervous system. Interestingly, SLITs are also known to protect pancreatic beta-cells from ER stress-induced apoptosis *via* the gradual release of Ca^2+^ from ER stores (58). Functionally, SLITs ligands along with ROBO receptors play an important role in attracting and repelling neuronal migration. This dual functionality is also observed in different cancers where SLITs can act both as a tumor suppressor and an oncogene. So far, the role of SLIT3 in PC progression and chemoresistance is unknown. Our study reveals that SLIT3 drives PC cell proliferation, stemness and migration (Fig. 6, *F*–*H*). Further, our data demonstrates that SLIT3 acts downstream of Orai3 and positively regulates chemoresistance, stemness, migration and invasion of gemcitabine-resistant cells (Fig. 7). Therefore, our study identifies SLIT3 and its crosstalk with Orai3 as a crucial driver of gemcitabine resistance in PC.

Interestingly, whole-genome studies on PC patient samples have revealed mutations in the SLIT and ROBO genes (38, 39, 40). These studies have highlighted a strong correlation between the dysregulated SLIT/ROBO signaling and early stages of pancreatic neoplastic transformation (39). The data from these studies strongly support the idea that aberrant regulation of axon guidance pathways is not merely a consequence but may actively drive the early events of tumor initiation and subsequent progression. Further, several independent studies have reported a crucial role of SLIT2, ROBO1 and ROBO2 proteins in PC initiation, progression and metastasis (39, 59, 60, 61, 62). Moreover, ROBO3 was shown to drive PC metastasis and chemoresistance using patient-derived xenografts and FACS-sorted PC biopsies (63). Notably, our unbiased transcriptomics data identified SLIT3, which was significantly decreased upon Orai3 silencing in GemR cells (Fig. 6*D*). Importantly, our earlier study has demonstrated that Orai3 is overexpressed in PC samples in comparison to healthy control pancreas and higher Orai3 expression is associated with poor prognosis (15). These observations provide a compelling mechanistic link, suggesting that Orai3 may serve as a critical upstream modulator that initiates activation of the SLIT/ROBO signaling pathway in PC progression. Thus, Orai3 may play a pivotal role in orchestrating the early molecular events that facilitate neoplastic transformation in pancreatic tissue, thereby contributing to PC progression, metastasis and chemoresistance.

NFAT transcription factor family has been widely studied in the context of immune cell function and disorders (64). Emerging literature reports elevated expression and enhanced transcriptional activity of NFAT isoforms in various cancers, which in turn augment the expression of genes driving tumorigenesis (65, 66). NFATc1-c4 are regulated by calcium-activated calcineurin and require sustained calcium signals, which are achieved through the opening of CRAC channels at the plasma membrane (67). In our study, we employed an unbiased bioinformatic approach to explore the relation of NFAT transcription factor family with gemcitabine resistance in PC (Fig. 8, *A* and *B*). We observed that NFATc1 overexpression increases SLIT3 expression and promoter activity, indicating that NFATc1 positively regulates SLIT3 transcription. Emerging evidence indicates that NFATc1 is the central regulator of tumor cell proliferation, survival, and metastatic progression (64, 65). An interesting study revealed contrasting roles for NFATc1 and NFATc2 in tumorigenesis, with NFATc2 functioning as a tumor suppressor and NFATc1 as an oncogene (68). In PC cells, NFATc1 drives proliferation and anchorage-independent growth by activating c-myc transcription (69). Our findings reveal that NFATc1 transcriptionallyregulates PC gemcitabine resistance by functioning as a bridge between Orai3 and SLIT3. Interestingly, our recent work demonstrated that NFATc1 plays an important role in regulating Orai3 expression in PC cells (70). Therefore, NFATc1, Orai3, and SLIT3 form intriguing feedforward loops that drive PC metastasis and chemoresistance.

Taken together, we reveal a crucial role of Orai3 in driving gemcitabine resistance. Further, we demonstrate that Orai3 regulates key features of chemoresistance, including enhanced proliferation, migration, invasion, stemness, and metastasis. Moreover, unbiased transcriptomics coupled with robust functional assays reveals that SLIT3 functions downstream of Orai3 to drive gemcitabine resistance. Finally, we show that NFATc1 drives SLIT3 transcription, thereby connecting Orai3 overexpression in GemR cells to SLIT3 augmentation (Fig. 9). Collectively, this study suggests that Orai3 inhibition can enhance the efficacy of chemotherapy regimens. Therefore, future studies are required to identify specific drug molecules that inhibit Orai3. Eventually, these drugs may be administered in combination with gemcitabine to improve its therapeutic usefulness. This approach would offer a novel way to overcome chemoresistance in pancreatic cancer.

**Figure 9:**
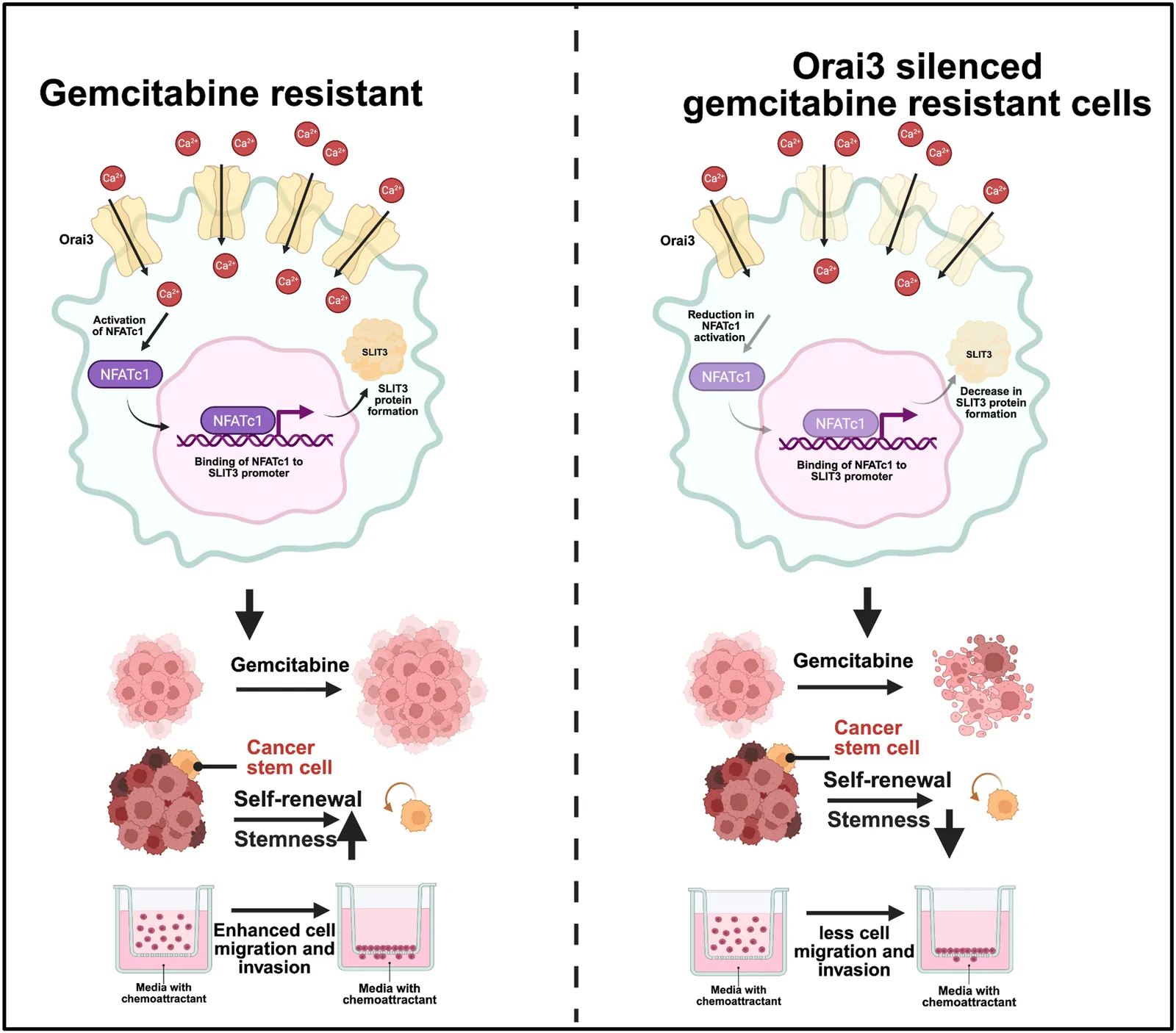
**Orai3 drives pancreatic cancer gemcitabine resistance *via* NFATc1-SLIT3 axis A graphical summary of our work demonstrates that Orai3 activates NFATc1, which in turn transcriptionally regulates the expression of SLIT3.** The NFATc1-SLIT3 axis functions in coordination to regulate gemcitabine resistance downstream of Orai3. Silencing of Orai3 reduces NFATc1 activation, thereby affecting SLIT3 transcription, which, in turn, disrupts chemoresistance and its key hallmarks, such as cell proliferation, migration, invasion, and stemness.

## Experimental procedures

### Cell culture

The cell line used in this study was obtained from the American Type Culture Center (ATCC). CFPAC-1 cells were grown in RPMI-1640 (Sigma) with 10% FBS, Panc-1 in DMEM high glucose (Sigma) with 10% FBS, and MiaPaCa-2 is also cultured in DMEM high glucose supplemented with 10% FBS and 2.5% horse serum (Himedia). Media used for culturing cells are also augmented with 1X anti-anti (Gibco) to prevent microbial contamination. Cells were maintained in a humidified chamber with 5% CO2 at 37 °C.

### Western blotting

The cells were washed with PBS (pH = 7.4) and trypsinised using 0.1% trypsin to make cell pellets. The suspension was lysed using NP40 (Thermo Fisher) and a protease inhibitor cocktail overnight at −80 °C. Protein lysates were collected by centrifugation at 13,000 rpm at 4 °C for 20 min. Protein samples were quantified using a Bicinchoninic acid kit (Thermo Scientific). The quantified samples were loaded onto different concentrations of acrylamide gels for separation, followed by wet electrophoretic transfer to polyvinylidene difluoride (PVDF) membranes (0.45 μm; Millipore). The membranes were blocked with 5% skimmed milk powder (Himedia) in Tris-buffered saline with 0.1% Tween (TBST) for 1 h at room temperature and probed with Orai3 antibody (1:500, Abcam), STIM1 antibody (1:500, BD biosciences), STIM2 (1:500, Abcam), Orai1 (1:500, Abcam), Orai2 (1:500, Abcam), E-cadherin (1:1000, Cell Signaling Technology), Vimentin (1:1000, Cell Signaling Technology), ALDH1A1 (1:1000, Cell Signaling Technology) and SLIT3 (1:100, SantaCruz) overnight at 4 °C. The blots were then incubated with horse-radish peroxidase (HRP) conjugated secondary rabbit or mouse antibody for 2 h at room temperature. The visualization was performed with ImageQuant LAS 4000 using a chemiluminescent substrate (Millipore).

### Lentiviral stable cell line generation

The stable Orai3 knockdown was established using human-specific shOrai3 and non-targeting control (shNT) sequences cloned into the lentiviral pGIPZ vector (Dharmacon), as described in our previously published study (71).

### RNA extraction

According to the manufacturer's protocol, total RNA was extracted from the cells using an RNeasy Mini Prep Kit (Qiagen). RNA was dissolved in 30 μl nuclease-free water. RNA extracted was quantified using a Nanodrop spectrophotometer (Thermo Fisher Scientific).

### RNA sequencing

Total RNA of different conditions, parental, GemR, shNT, and shOrai3 GemR, were sent to Clevergene, Bengaluru (India) for RNA-sequencing. The sequenced data was generated using Illumina NovaSeq. The data quality was assessed using FastQC and MultiQC software. The quality control-approved reads were aligned to the indexed human genome (GRCh38.p14) utilizing the STAR v2 aligner. Gene-level expression values were derived as read counts using featureCounts software. The DEseq2 package was used to analyze differential expression, and normalized counts were obtained. Data quality was assessed using FastQC and MultiQC software. GRCm39 was used as the reference genome. Normalized gene expression values were obtained from raw read counts using feature counts software. The differentially expressed genes were screened using log2FC of +2 and −2 for upregulated and downregulated genes, respectively. These genes were then analyzed for pathway enrichment analysis using GO pathway analysis software, and a bubble plot was generated to represent the enriched pathways. Common genes that were differentially regulated in GemR *versus* parental and shOrai3 *versus* shNT were identified using the Venny 2.0 tool.

### Real-time PCR

Extracted RNA was reverse transcribed to generate first-strand cDNA (High-Capacity cDNA Reverse Transcription Kit, ThermoFisher Scientific). Quantitative real-time PCR was performed using TB Green Premix Ex Taq II (SYBR Green, Takara) according to the manufacturer’s instructions on a QuantStudio 6 Flex real-time PCR system (Thermo Fisher Scientific). The primers for different genes are listed in Table 1.

**Table 1:** qPCR primer list

Gene name	Primer sequence (5′—> 3′)
SLIT3 FP	GAGAGGGAGCTTTCGATGGA
SLIT3 RP	GTCATTACTCACACAGCCGATCA
CD44 FP	GCAACTGAGACAGCAACCAAG
CD44 RP	GGTGTGGTTGAAATGGTGCTG
CD24 FP	GCTCCTACCCACGCAGATT
CD24 RP	GGTGGTGGCATTAGTTGGAT
BMI1 FP	ATCCTTCTGCTGATGCTGCC
BMI1 RP	TGCATCACAGTCATTGCTGC
EPCAM FP	GTGCTGGTGTGTGAACACTG
EPCAM RP	TCCTTCTGAAGTGCAGTCCG

### SLIT3 promoter cloning

The human Slit3 promoter was obtained from the Eukaryotic Promoter Database (EPD), and the sequence was analyzed using NCBI-BLAST to find the mRNA start site and the first codon. Primers were designed to amplify the 991 bp region (−969 to +23 w.r.t start codon) of the Slit3 core promoter. PCR amplification of the Slit3 promoter was done using Phusion High Fidelity Polymerase (F503, Thermo Fisher Scientific) and the PCR amplicon was cloned into psiCHECK-2 dual-luciferase reporter vector (C8021, Promega) at the SacII/MluI sites. The clones were verified through restriction digestion. The primers used for cloning are listed in Table 2.

**Table 2:** SLIT3 cloning primers

Promoter	Primer sequence (5′—> 3′)
SacII_SLIT3p_FP	TAA GCA GAG CTC ATG GCC CCC GGG TGG GCA G
MluI_SLIT3p_RP	TGC TTA GGT ACC TTA GGA ACA CGC GAG GCA GCC G

### siRNA transfections

For siRNA-mediated transfections, cells were seeded in 6-well plates and transfected using the JetPrime reagent (Sartorius) according to the manufacturer's instructions. To validate the knockdown efficiency, cells were harvested 48 h post-transfection. The sequences of human siOrai3 (Eurogentec), siSLIT3 (Eurogentec), siNFATc1 (Eurogentec), and non-targeting control (Eurogentec) are provided in Table 3.

**Table 3:** siRNA sequences

siRNA	Sequence
h siOrai3	GGGUCAAGUUUGUGCCCAU
h siSLIT3	GAGCUAUGCGACGAGGUGA
siNT (non-targeting pool)-1	UGGUUUACAUGUCGACUAA
siNT (non-targeting pool)-2	UGGUUUACAUGUUGUGUGA
siNT (non-targeting pool)-3	UGGUUUACAUGUUUUCUGA
siNT (non-targeting pool)-4	UGGUUUACAUGUUUUCCUA
h siNFATc1-1	UCAGAAACUCCGACAUUGA
h siNFATc1-2	UUUCGGAAUCAGAGGAUAA
h siNFATc1-3	GGACAGCUAUCCGGUCGUG
h siNFATc1-4	AGGAAGAACACACGGGUAC

### Luciferase assay

CFPAC-1 GemR cells were seeded in a 24-well plate at a density of 50,000 cells/well. Cells were co-transfected with SLIT3p and siNT/siNFAT2 using a JetPrime transfection reagent (101000046, Sartorius). After 48 h of transfection, cells were assayed for luciferase activity using the Dual-Luciferase Assay kit (E1910, Promega) according to the manufacturer’s protocol. Renilla luciferase was used for normalization.

### Cell cycle analysis

To assess cell cycle progression, pancreatic cancer cells were seeded in a 6-well plate and treated with 5 μM gemcitabine the following day. After 72 h of treatment, cells were harvested and kept in 70% ethanol for 24 h at −20 °C. The next day, cells were treated with RNase at 37 °C for 1 h, then stained with Propidium Iodide (PI) in the dark for 30 min. The cell cycle distribution was detected using BD FACSVerse Cell Analyzer (BD Bioscience).

### Ca^2+^ imaging

Pancreatic cancer cells were grown in confocal dishes (200350, SPL life sciences) for Ca^2+^ imaging experiments. Ratiometric dye Fura-2 AM (F1221, Invitrogen) was a Ca^2+^ indicator. Cells were loaded with Fura-2 AM for 30 min at 37 °C and 5% CO2 in the corresponding media. The detailed experimental protocol was followed, as reported previously (71, 72).

### Apoptotic assay

CFPAC-1 cells were seeded in a 24-well plate and treated with different concentrations of gemcitabine for 72 h. Cells were stained with Annexin V – TRITC labelled (AAT Bioquest) and DAPI to measure the cell death using BD FACSVerse Cell Analyzer (BD Bioscience).

### Spheroid formation assay

Spheroids were generated using the hanging drop method. A drop of 10 μL of complete media with 40,000 cells was pipetted onto the lid of 100 mm Petri dishes and was inverted over the dish containing PBS to avoid drying. Dishes were stored at 37 °C for 4 days for spheroids to form. After the period of incubation, images were taken using a Nikon stereo zoom microscope, and the area of spheroids was calculated using NIS Elements D version 5.21.03 software.

### Migration assay

To study migration assay, we used Boyden chamber transwells (3422, Corning). Different groups (Parental, GemR, shNT, and shOrai3 GemR) of CFPAC-1 cells (1 × 10ˆ5) and AsPC-1 cells (80 × 10ˆ3) were seeded in the medium containing 3% FBS and 1% FBS in the upper chambers of the 24-well plate, respectively. The lower chambers were filled with 650 ul of medium containing 10% FBS. The cells were incubated for 48 h at 37 °C. After the incubation period, cells were fixed with 3.7% formaldehyde, permeabilized using 100% methanol, and stained with 1% solution of crystal violet. The migrated cells were counted using ImageJ software.

### Cell proliferation assay

Cell proliferation was assessed using a BrdU-based colorimetric immunoassay kit (11647229001, Roche). 5 × 10ˆ3 cells were seeded for different groups (Parental, GemR, shNT, and shOrai3 GemR) in 96-well plates, and the proliferation rate was measured at various time points. The steps in quantifying cell proliferation were followed as per the protocol provided with the kit.

### Phalloidin staining

For actin filament visualization, cells were grown on a glass coverslip. After 24 h, cells were washed thrice with chilled PBS, then fixed on ice with 3.75% formaldehyde solution in PBS for 15 min. Following fixation, cells were permeabilized with 200 μl 0.5% Triton X-100 at room temperature for 10 min. Blocking was done using 1% BSA in PBS at 4 °C for 30 min. Cells were stained with Alexa Fluor 594 Phalloidin (Thermofisher Scientific, A12381) for 20 min at room temperature. After staining, coverslips were air-dried, followed by mounting with SlowFade Gold Antifade Mountant with DAPI (ThermoFisher Scientific, S36938) and imaged using a confocal microscope. F-actin distribution was quantified using ImageJ software, according to the published methodology (73).

### Zebrafish husbandry

The zebrafish used in this study were housed at the Regional Center for Biotechnology, India. The zebrafish studies were performed in compliance with the ethical protocols approved by the Institutional Animal Ethics Committee (Approval no. RCB/IAEC/2022/142).

### Zebrafish xenograft microinjection

Microinjections were performed using the human pancreatic cancer cell line CFPAC-1(Parental, GemR, shNT GemR and shOrai3 GemR). Cells were stained with lipophilic Vybrant CM-DiI (ThermoFisher Scientific) at a 4 μl/ml concentration. The staining procedure was performed as per the manufacturer’s instructions. Cells were resuspended to a final concentration of 30 × 10^3^/μl in 1X Versene (0.2 g EDTA(Na4) per liter of Phosphate Buffered Saline) before injection to avoid capillary clogging. The labelled tumor cells were microinjected into zebrafish embryos using borosilicate glass capillaries under a fluorescence stereomicroscope (Nikon, SMZ800N) equipped with a FemtoJet 4i microinjector (Eppendorf). No immune suppression was required for transplantation of human cells into zebrafish, as during early embryonic development, specifically from the blastula stage to 48 hpf, the adaptive immune system remains immature, eliminating the need for immune suppression during the procedure (17, 74). Cells were injected into the perivitelline space (PVS) of 2-days-post-fertilization (dpf) zebrafish embryos, which were pre-anesthetized with 1X Tricaine (Sigma-Aldrich). Following injection, the zebrafish xenografts were transferred to E3 medium and incubated at 34 °C. At 1-h post-injection (hpi), the xenografts were examined for the presence or absence of cells in circulation, and those with severe oedema, cells in the yolk sac, cell debris, or noninjected were excluded (75). At four dpi, xenografts were anaesthetized to image the metastatic cells in caudal hematopoietic tissue (CHT). Xenografts were kept in 1X Phenylthiourea (PTU) to suppress pigmentation until the experiment was completed.

### Statistical analysis

All experiments were performed in three or more independent biological replicates. Data shown as mean ± SEM. A one-sample *t* test and an unpaired Student’s *t* test were performed to determine the statistical significance. A one-way ANOVA was performed for data containing more than two conditions. *p* < 0.05 was considered as significant and is presented as ‘‘∗’’, *p* < 0.01 is presented as ‘‘∗∗’’, *p* < 0.001 is presented as “∗∗∗” and *p* < 0.0001 is presented as “∗∗∗∗”.

## Data availability

All relevant data are within the paper and in supplementary files. The raw RNA sequencing data have been deposited in INSDC (International Nucleotide Sequence Database Collaboration) under the accession number PRJINA00728.

## Supporting information

This article contains supporting information.

## Conflict of interest

The authors declare that they have no conflicts of interest with the contents of this article.

## Figures and Tables

**Supplementary Figure S1:**
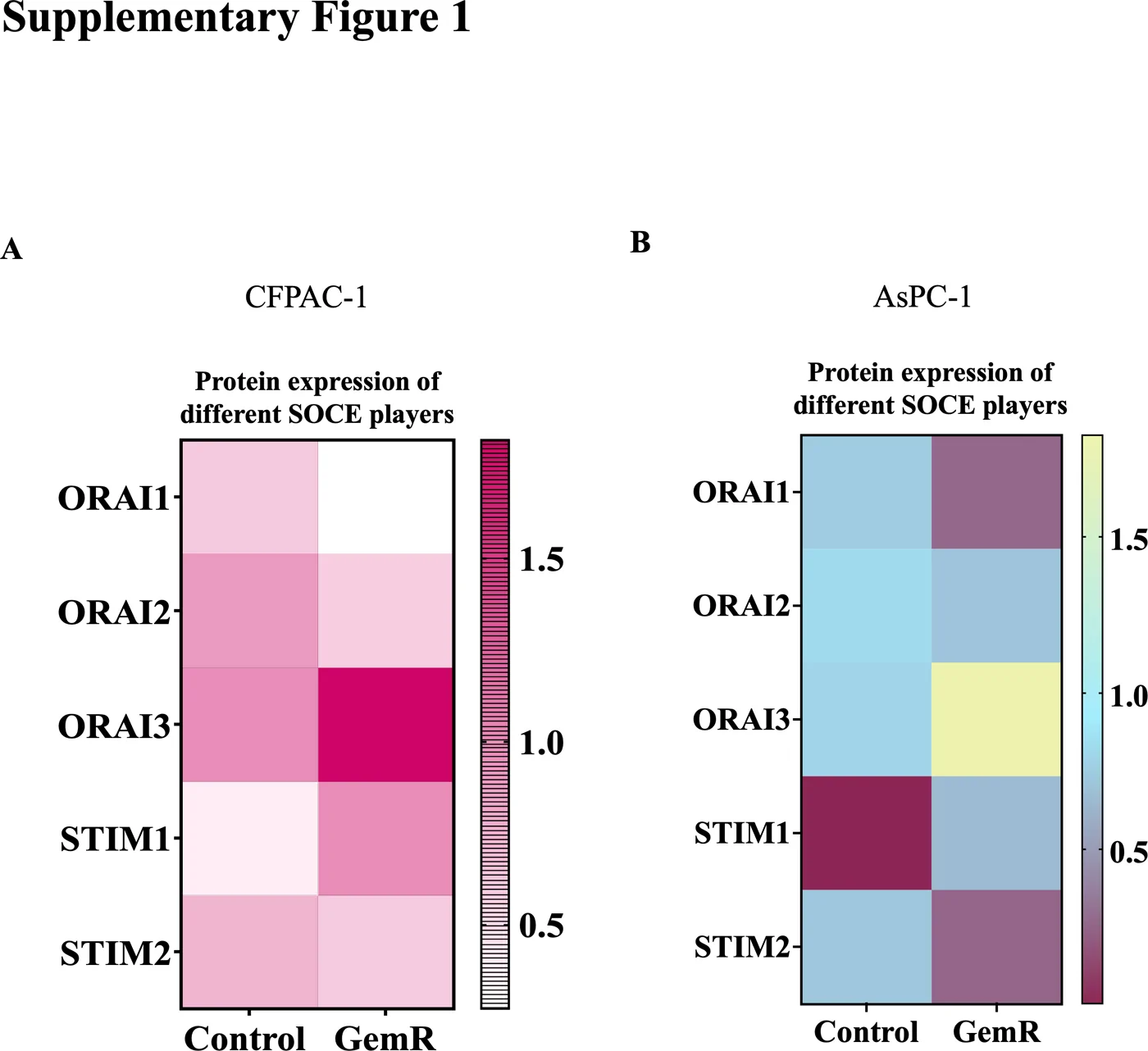


**Supplementary Figure S2:**
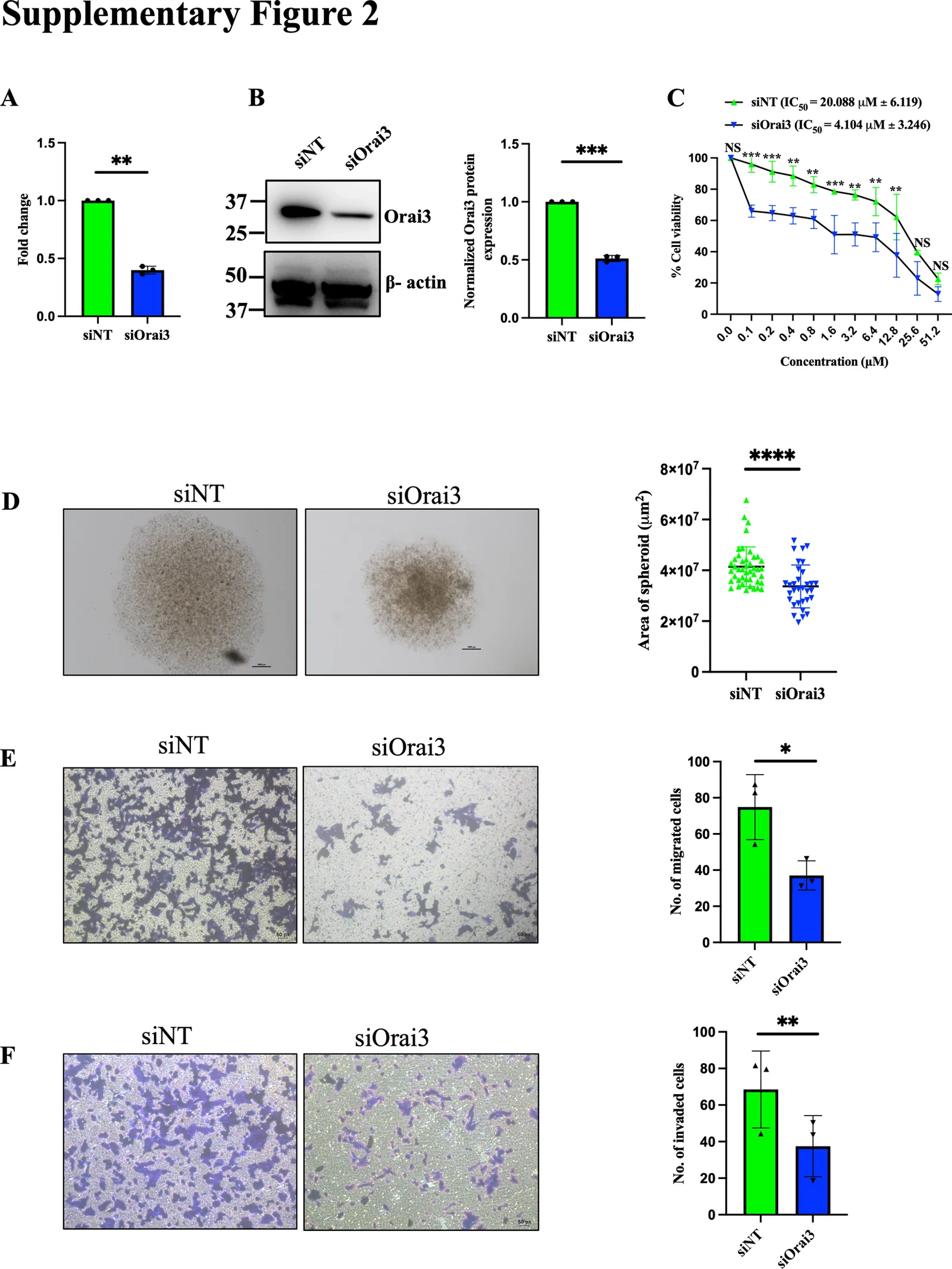


**Supplementary Figure S3:**
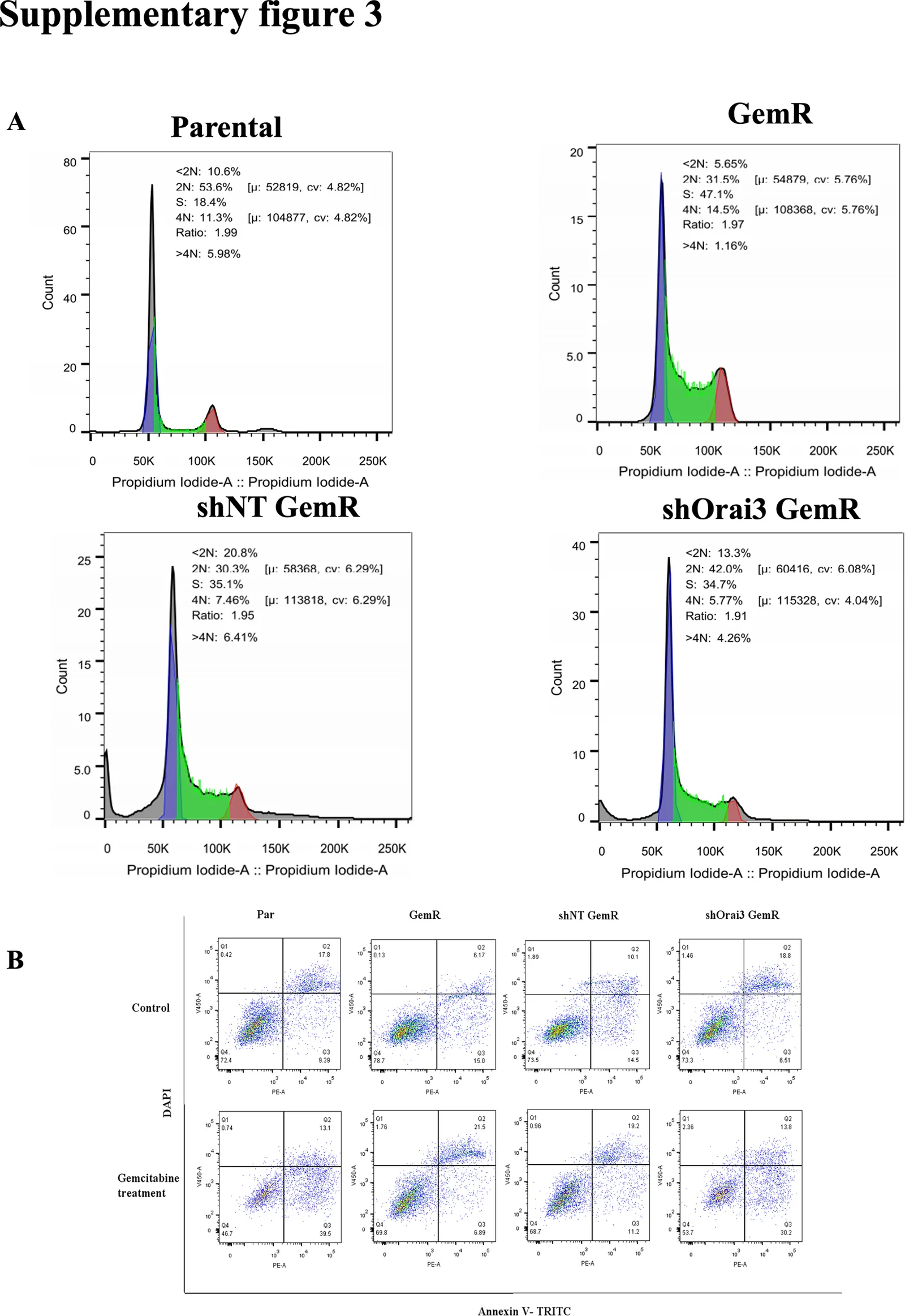


**Supplementary Figure S4:**
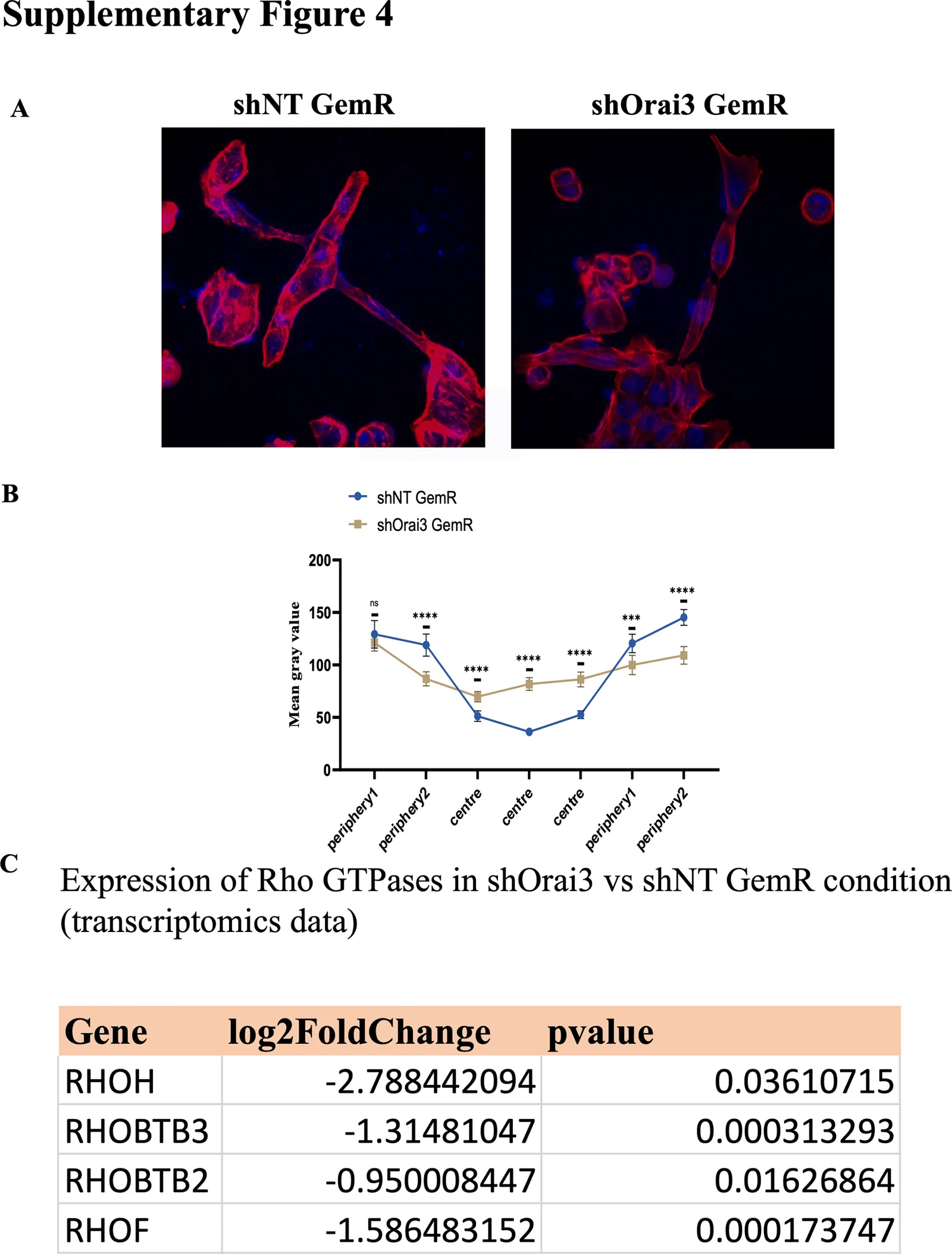


**Supplementary Figure S5:**
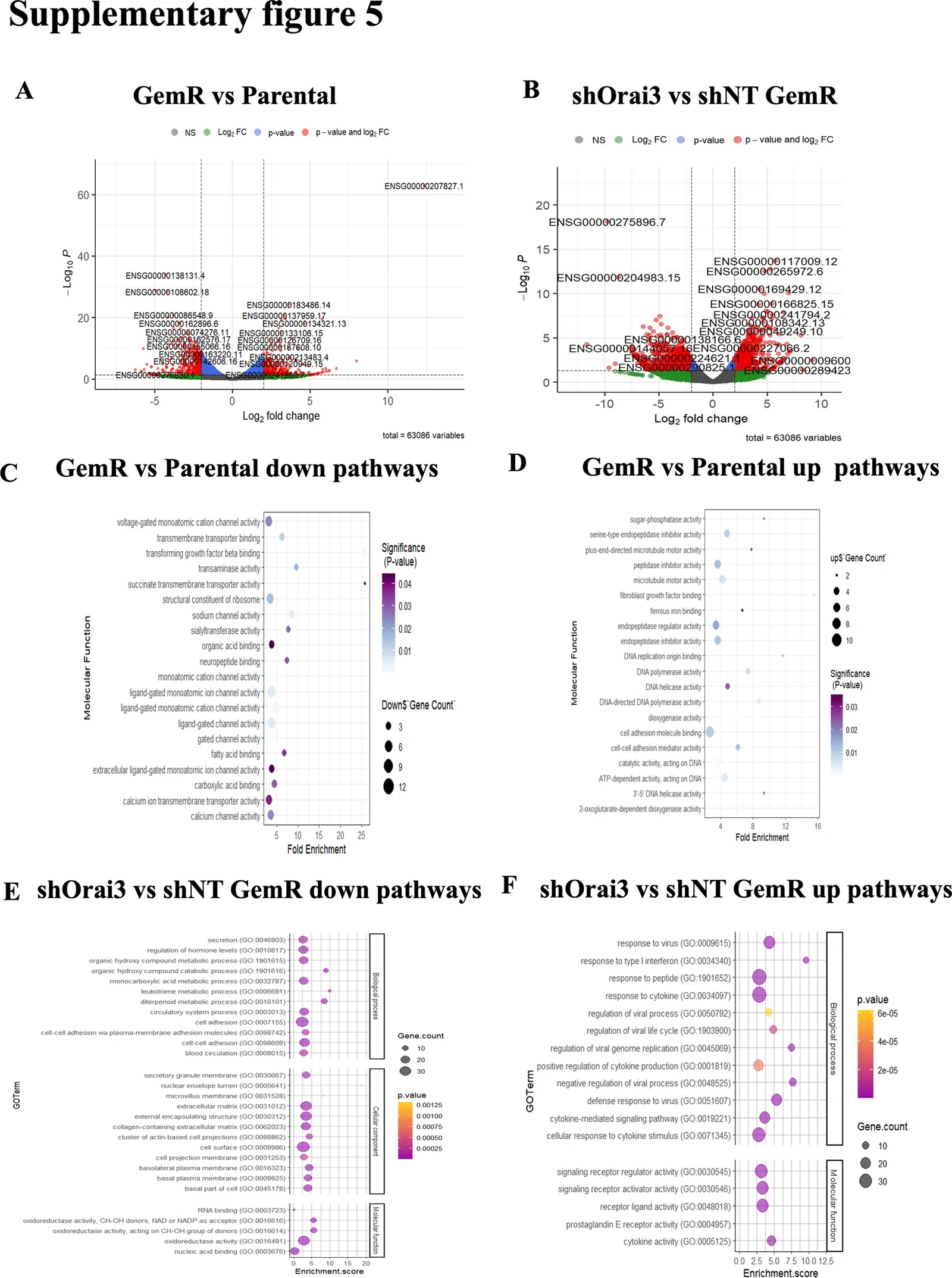


**Supplementary Figure S6:**