# Supplementary material for: The Ca2+ channel Orai3 drives gemcitabine resistance in pancreatic cancer through NFATc1-dependent SLIT3 expression
Source: J Biol Chem. 2026 Aug 25;302(10):113480. doi: 10.1016/j.jbc.2026.113480 (PMC13625800; doi:10.1016/j.jbc.2026.113480)
Supplement: Supplementary file 1 [file mmc2.docx]

**Supplementary Figure 1: Heatmap showing expression of various SOCE players in CFPAC-1 and AsPC-1 Control and GemR cells.**

**A)** Expression of different SOCE players in CFPAC-1 Control and GemR.

**B)** Expression of different SOCE players in AsPC-1 Control and GemR

**Supplementary Figure 2: Transient silencing of Orai3 regulates gemcitabine resistance *in vitro.***

**A)** Validation of Orai3 mRNA expression in CFPAC-1 parental vs GemR (N=3). ***P =* 0.0010

**B)** Representative western blot confirming siRNA-based silencing of Orai3 in CFPAC-1 GemR cells. Densitometric analysis of Orai3 knockdown using ImageJ is shown graphically (N=3). ***P =* 0.0010

**C)** MTT assay shows the change in the chemosensitivity of GemR cells upon transient silencing Orai3 in CFPAC-1 GemR (N=3). ****P* = 0.0001, ****P* = 0.0007, ***P* = 0.0011, ***P* = 0.0070, ****P* = 0.0004, ***P* = 0.0013, ***P* = 0.0045, ***P* = 0.0020

**D)** Spheroid formation assay depicting a spheroid's size change on transient silencing Orai3 in CFPAC-1 GemR cells. Each spheroid was defined as a region of interest (ROI), and its area was measured using Nikon software. The quantified area of spheroids is represented in the graph (N=3). *****P* <0.0001

**E)** Boyden chamber transwell assay for studying the migration potential of GemR cells upon transient silencing of Orai3. Graph showing a quantitative analysis of migration potential in Orai3-knockdown cells (N=3). **P* = 0.0292

**F)** Transwell assay for measuring invasion potential upon transient silencing of Orai3 in CFPAC-1 GemR cells and quantification of the number of cells that invade the Matrigel coating on the transwell due to the FBS gradient is shown graphically. Images were captured using a Nikon bright-field microscope at 10x (N=3). ***P* = 0.0092

Data presented are mean ± SD. One-sample t-test was performed for panels A and B, an unpaired student t-test was performed for panels D and E, a two-way ANOVA followed by Šídák's multiple comparisons test was performed for panel C, and a paired t-test was done for panel F using GraphPad Prism software. Here, * *p* <0.05; ** *p* < 0.01; *** *p* < 0.001 and **** *p* < 0.0001.

**Supplementary Figure 3:** **Orai3 regulates G1 to S transition and apoptosis of CFPAC-1 GemR cells.**

**A)** Representative graphs showing different stages of the cell cycle in parental, GemR, and shNT GemR and shOrai3 GemR CFPAC-1 cells.

**B)** Representative quadrant plots showing apoptosis of parental, GemR, and shNT GemR and shOrai3 GemR CFPAC-1 cells when treated with gemcitabine.

**Supplementary Figure 4: Orai3 regulates actin dynamics.**

**A)** Representative microscopic images showing the distribution of actin in shNT and shOrai3 GemR CFPAC-1 cells

**B)** Quantitation of mean gray value corresponding to actin distribution in the shNT and shOrai3 GemR. *****P* <0.0001, ****P* = 0.002285

**C)** Expression of Rho GTPases in shOrai3 vs shNT GemR CFPAC-1 cells.

**Supplementary Figure 5: RNA-seq data analysis.**

**A)** Volcano plot showing differentially expressed genes in GemR vs parental.

**B)** Volcano plot showing differentially expressed genes in shOrai3 GemR vs shNT GemR.

**C)** GO pathway enrichment analysis of downregulated genes in GemR vs Parental conditions.

**D)** GO pathway enrichment analysis of upregulated genes in GemR vs Parental conditions.

**E)** GO pathway enrichment analysis of downregulated genes in shOrai3 GemR vs shNT GemR conditions.

**F)** GO pathway enrichment analysis of upregulated genes in shOrai3 GemR vs shNT GemR conditions.

**Supplementary Figure 6: NFATc1 expression upon silencing of Orai3 in CFPAC-1 GemR.**

**A)** Representative Western blot and densitometry showing NFATc1 expression on silencing Orai3 in CFPAC-1 GemR (N=3). ***P* = 0.0019

Data presented are mean ± SD. A one-sample t-test was performed for panel A using GraphPad Prism software. Here, * *p* <0.05; ** *p* < 0.01; *** *p* < 0.001 and **** *p* < 0.0001.
